# IL-33 is associated with alveolar dysfunction in patients with viral lower respiratory tract disease

**DOI:** 10.1016/j.mucimm.2024.12.001

**Published:** 2025-04

**Authors:** Ian C. Scott, Natalie van Zuydam, Jennifer A. Cann, Victor Augusti Negri, Kalliopi Tsafou, Helen Killick, Zhi Liu, Christopher McCrae, D. Gareth Rees, Elizabeth England, Molly A. Guscott, Kirsty Houslay, Dominique McCormick, Anna Freeman, Darren Schofield, Adrian Freeman, E. Suzanne Cohen, Ryan Thwaites, Zach Brohawn, Adam Platt, Peter J.M. Openshaw, Malcolm G. Semple, J. Kenneth Baillie, Tom Wilkinson, the ISARIC4C Investigators

**Affiliations:** a Translational Science and Experimental Medicine, Research and Early Development, Respiratory and Immunology, BioPharmaceuticals R&D, AstraZeneca, Cambridge, UK; b Discovery Sciences, Research and Early Development, BioPharmaceuticals R&D, AstraZeneca, Cambridge, UK; c Clinical Pharmacology and Safety Sciences, Research and Early Development, BioPharmaceuticals R&D, AstraZeneca, Gaithersburg, MD, USA; d Translational Sciences and Experimental Medicine, Research and Early Development, Respiratory and Immunology, BioPharmaceuticals R&D, AstraZeneca, Gaithersburg, MD, USA; e Biologics Engineering, Research and Early Development, Oncology R&D, AstraZeneca, Cambridge, UK; f Bioscience Asthma and Skin Immunity, Research and Early Development, Respiratory and Immunology, BioPharmaceuticals R&D, AstraZeneca, Cambridge, UK; g Department of Clinical Infection, Microbiology, and Immunology, Institute of Infection, Veterinary and Ecological Sciences, University of Liverpool, Liverpool, UK; h School of Clinical and Experimental Sciences, Faculty of Medicine, University of Southampton, Southampton General Hospital and University Hospital Southampton NHS Foundation Trust, Southampton, UK; i National Heart and Lung Institute, Imperial College London, London, UK; j Baillie Gifford Pandemic Science Hub, University of Edinburgh, Edinburgh, UK

**Keywords:** Aerocyte, Alarmin, Alveolar, COVID-19, IL-33, Viral LRTD

## Abstract

Interleukin (IL)-33 is released following tissue damage, causing airway inflammation and remodelling via reduced IL-33 (IL-33^red^)/serum stimulation-2 (ST2) and oxidised IL-33 (IL-33^ox^)/receptor for advanced glycation end products (RAGE)/epidermal growth factor receptor (EGFR) pathways. This study aimed to identify associations of IL-33 with clinical outcomes and pathological mechanisms during viral lower respiratory tract disease (LRTD). Ultra-sensitive immunoassays were developed to measure IL-33^red^, IL-33^ox^ and IL-33/sST2 complexes in samples from patients hospitalised with COVID-19. Immunohistochemistry and multiomics were used to characterise lung samples. Elevated IL-33 in the airway and IL-33/sST2 complex in the circulation correlated with poor clinical outcomes (death, need for intensive care or mechanical ventilation). IL-33 was localised to airway epithelial and endothelial barriers, whereas *IL1RL1* was expressed on aerocytes, alveolar endothelial cells specialised for gaseous exchange. IL-33 increased expression of mediators of neutrophilic inflammation, immune cell infiltration, interferon signalling and coagulation in endothelial cell cultures. Endothelial IL-33 signatures were strongly related with signatures associated with viral LRTD. Increased IL-33 release following respiratory viral infections is associated with poor clinical outcomes and might contribute to alveolar dysfunction. Although this does not show a causal relationship with disease, these results provide a rationale to evaluate pathological roles for IL-33 in viral LRTD.

## Graphical abstract

Hypothesis for the pathological roles of IL-33 in viral LRTD.

(a) Respiratory viral infections can damage the airway epithelium and cause release of IL-33^red^.

(b) IL-33^red^ can activate the ST2 receptor on specialised alveolar capillary endothelial cells called aerocytes.

(c) IL-33-driven activation of aerocytes can initiate alveolar dysfunction, including inflammation, immune cell infiltration, coagulation, and oedema to disrupt gaseous exchange and increase hypoxia that potentially could lead to acute respiratory failure (region shown as magnified image).

(d) IL-33 can be oxidised and signal via RAGE/EGFR on alveolar AT1 epithelial cells and airway remodelling in response to viral infection that could contribute to viral LRTD.

**Figure:**
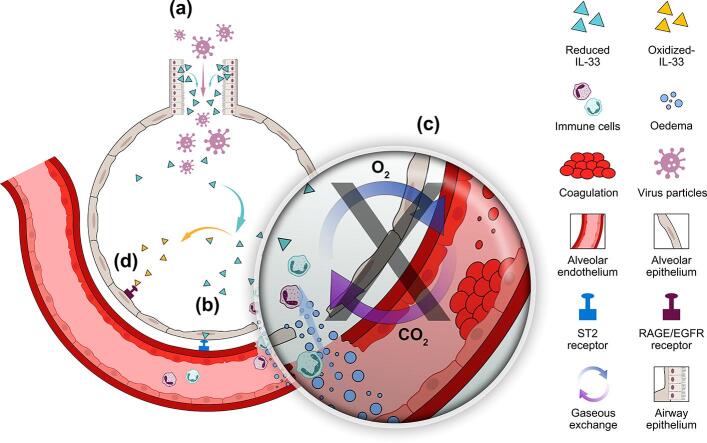

## Introduction

Interleukin (IL)-33 is an IL-1 family cytokine that initiates immune responses following exposure to environmental irritants, trauma or infection.^1^ However, elevated IL-33 can cause inflammation, resulting in tissue damage and remodelling, which has been shown to contribute to respiratory diseases such as asthma and chronic obstructive pulmonary disease (COPD).^2^, ^3^ The role of IL-33 during infection remains poorly understood, but potentially includes both innate and adaptive immune responses during host defence^4^, ^5^, ^6^, ^7^ and infectious diseases.^8^, ^9^, ^10^ Animal and human models of respiratory viral exacerbations suggest that IL-33 can amplify type I and II inflammation, modify the expression of the IL-33 receptor, serum stimulation-2 (also known as ST2, ST2L, IL-1 receptor-like 1 [IL1RL1]) and increase airway hyperresponsiveness.^11^, ^12^, ^13^ However, there is limited understanding of the clinical and mechanistic associations of IL-33 in respiratory viral infections that lead to viral lower respiratory tract disease (LRTD).

IL-33 is a constitutively expressed protein localised in the nucleus of structural cells with highest levels stored in epithelial and endothelial cells in barrier tissues.^1^, ^14^, ^15^ Pre-stored reduced IL-33 (IL-33^red^) is rapidly released from cells during necrosis or tissue injury as full length or processed forms that signal through a receptor complex of ST2 and the IL-1 receptor accessory protein.^16^, ^17^ IL-33^red^ can be rapidly oxidised, through two intramolecular disulphide bonds, whereas oxidised IL-33 (IL-33^ox^) signals via the receptor for advanced glycation end products (RAGE)/epidermal growth factor receptor (EGFR) complex to modulate epithelial remodelling and mucus secretion.^18^, ^19^ Soluble ST2 (sST2), a secreted variant of ST2, is a decoy receptor for IL-33.^18^ IL-33 is predominantly released from epithelial and endothelial barriers.^1^ However, understanding of the different forms of IL-33 in tissues is lacking because selective assays for IL-33^red^, IL-33^ox^ and IL-33/sST2 complex have not been available.^20^ The ST2 receptor and sST2 are typically expressed by immune and endothelial cells (ECs).^1^ However, the roles of IL-33 in ECs are relatively poorly understood.^21^ Circulating sST2 levels are elevated during the course of inflammatory and infectious disease and elevated levels of sST2 are associated with poor clinical outcomes.^22^.

This study aimed to identify the associations between IL-33 and poor clinical outcomes (defined as death or the need for admission to an intensive care unit or mechanical ventilation) and disease mechanisms in patients hospitalised with coronavirus disease 2019 (COVID-19) and potentially other viral LRTD.

## Results

### Demographics and clinical characteristics of the Southampton and ISARIC4C cohorts of patients hospitalised with COVID-19

Clinical and laboratory data were collected from 100 patients in the Southampton cohort and 182 patients in the ISARIC4C cohort who were hospitalised with COVID-19 (Supplementary Tables 1 and 2). Patient treatments and some outcome analyses were reported previously.^23^, ^24^ Patients were predominantly white, male and non-smokers, with mean ages of 62 and 61 years in the Southampton and ISARIC4C cohorts, respectively. Common comorbidities in both cohorts included chronic respiratory and cardiovascular diseases. Patients in the Southampton cohort had an overall mortality of 17 %, and 44 % of patients met the composite clinical endpoint of either death within 30 days, admission to the ICU or the requirement for invasive mechanical ventilation. Patients in the ISARIC4C cohort had an overall mortality of 15 %, with 25 % of patients meeting the composite clinical endpoint.

### Development of immunoassays for the measurement of IL-33 forms in clinical samples

Commercial IL-33 immunoassays, Pro Th17 (Bio-Plex) and Duoset ELISA (Biotechnie), detected all forms of IL-33 with differential sensitivities, whereas the Mesoscale Discovery (MSD) assay only detected IL-33^red^ with a lower limit of quantification (LLOD) of 0.59 pg/ml (Supplementary Fig. 1a–c, respectively). These results, alongside other reports, failed to identify commercial assays selective for IL-33^red^, IL-33^ox^ and IL-33/sST2 complex.^16^, ^25^, ^26^ Novel ultra-sensitive immunoassays were developed for selective measurement of IL-33^red^, IL-33^ox^ and IL-33/sST2 complex in clinical samples (Supplementary Figs. 2 and 3, and Supplementary Tables 3 and 4). Performance of these assays was characterised by dilution linearity, parallelism and spike recovery in serum, plasma and nasal MLF, and by analyte selectivity (Supplementary Fig. 3 and Supplementary Table 5). LLOD and upper limit of detection (ULOD) and quantification (LLOQ, ULOQ), and minimal required dilution (MRD) in serum, plasma and nasal MLF were determined and are summarised in Supplementary Table 6.

## IL-33 release is increased in patients hospitalised with COVID-19

IL-33/sST2 complex, but not IL-33^red^ or IL-33^ox^, was detected in serum and plasma from healthy participants and in patients at the time of hospitalisation with COVID-19 (Fig. 2a, Supplementary Fig. 4). Levels of IL-33/sST2 complex and sST2 (free or complexed with IL-33^red^, Supplementary Fig. 5) were elevated in patients with COVID-19 compared with healthy participants (Fig. 2a). In patients hospitalised with COVID-19, levels of IL-33/sST2 complex and sST2 were poorly correlated (Supplementary Fig. 6). IL-33^red^ and IL-33^ox^ were detected in nasal MLF samples with median IL-33^ox^ levels increased 14-fold (*p* < 0.0001), whereas IL-33^red^ were not increased (*p* = 0.7891), in patients hospitalised with COVID-19 compared with healthy participants (Fig. 2b). In healthy participants, levels of sST2 in nasal MLF samples were significantly lower than in serum samples (119-fold lower, *p* < 0.0001), (Supplementary Fig. 7). Nasal IL-33^red^ and IL-33^ox^ correlated strongly with each other but poorly with plasma IL-33/sST2 complex (Supplementary Fig. 8a–c). In summary, different forms of IL-33 were detected in the airway and the circulation (summarised in Fig. 2c). Circulating and airway forms of IL-33 were significantly elevated in patients with COVID-19 compared with healthy participants, indicating potential roles of IL-33 in COVID-19.

**Fig. 1:**
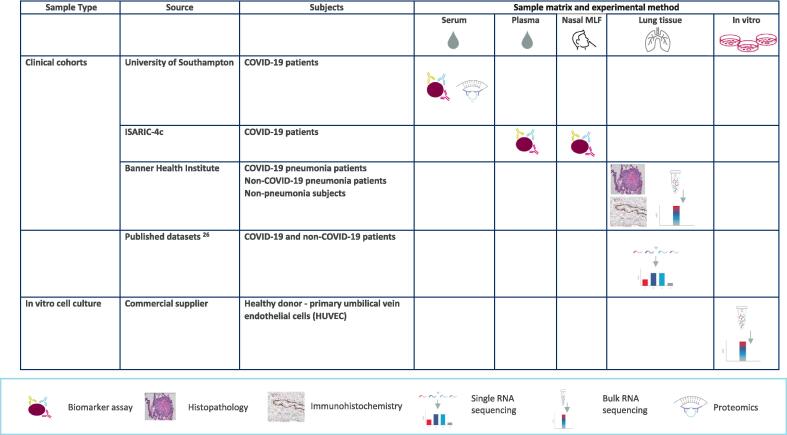
Summary of samples and experimental methods used in this study. COVID-19 = coronavirus disease 2019; MLF = mucosal lining fluid.

**Fig. 2:**
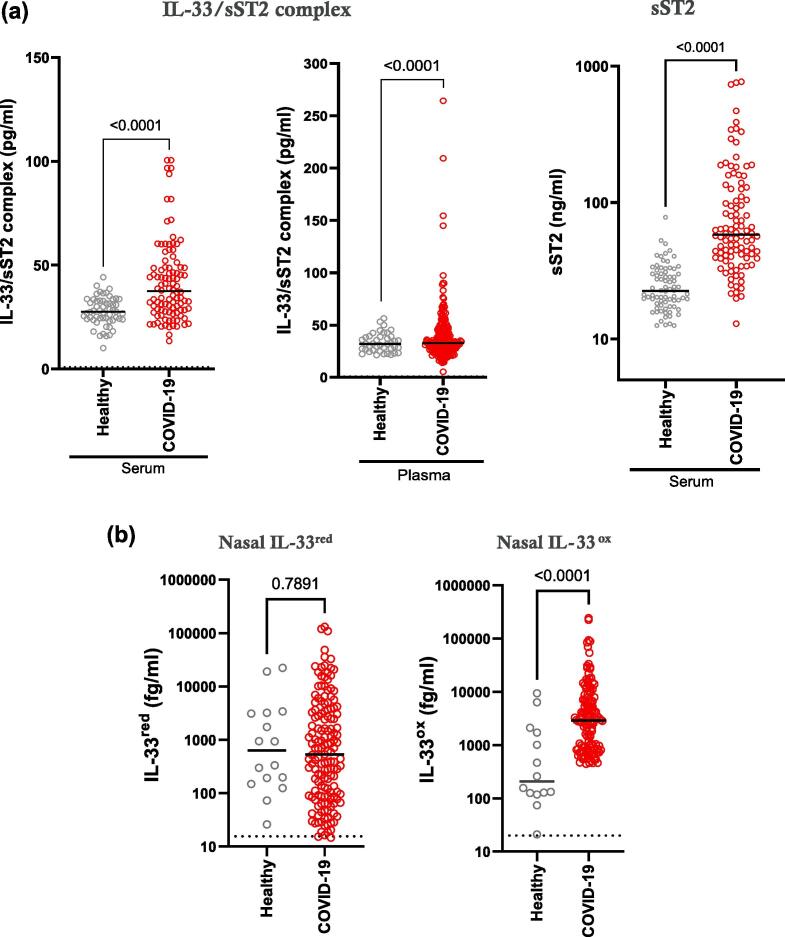

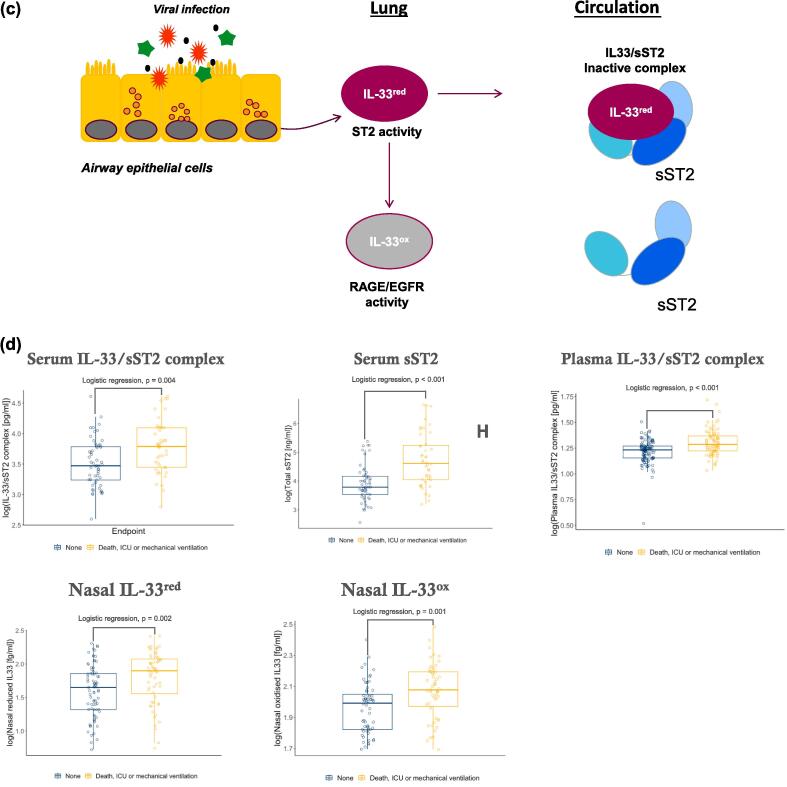

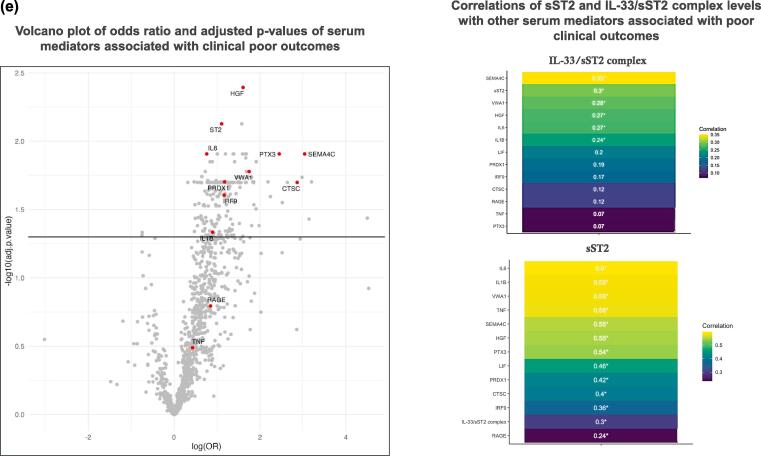
IL-33 and sST2 release are elevated in patients with COVID-19 and are associated with poor clinical outcomes. (A) measurement of IL-33/sST2 complex in serum (left panel) and plasma (middle panel) from healthy individuals (serum, *n* = 56; plasma, *n* = 40) and patients hospitalised with COVID-19 (serum, *n* = 100; plasma, *n* = 203). Measurement of serum sST2 (right panel) from healthy individuals (*n* = 76) and patients hospitalised with COVID-19 (*n* = 100). (B) measurement of IL-33^red^ (left panel) and IL-33^ox^ (right panel) in nasal mucosal lining fluids from healthy individuals (*n* = 16 and 14, respectively) and patients with COVID-19 (*n* = 151 and 130, respectively). Individual patient data points are shown. Median bar, *p* values are shown (Mann Whitney test) (A and B) (C) Schematic diagram of the different forms of IL-33 in the lung and in the circulation. IL-33^red^ released from virally infected airway epithelial cells can be rapidly oxidized (IL-33^ox^) locally. IL-33^red^ released into the circulation forms an inactive complex with sST2 (IL-33/sST2 complex) (D) measurement of serum IL-33/sST2 complex, serum sST2, plasma IL-33/sST2 complex, nasal IL-33^red^ and nasal IL-33^ox^ in patients who did or did not meet the composite clinical endpoint. (E) volcano plot (left panel) of associations of serum proteins with poor clinical outcomes adjusted for age, gender and ethnicity; the horizontal line represents adjusted *p* < 0.05; selected proteins are highlighted by red dots. Heat maps (right panel) showing the correlations between IL-33/sST2 complex and sST2 levels with selected other serum mediators; r values are shown; *adjusted *p* values < 0.05. COVID-19 = coronavirus disease 2019; IL = interleukin; IL-33^ox^ = oxidised IL-33; IL-33^red^ = reduced IL-33; ns = not significant; OR = odds ratio; r = correlation coefficient; sST2 = soluble serum stimulation-2. (For interpretation of the references to colour in this figure legend, the reader is referred to the web version of this article.)

**Fig. 3:**
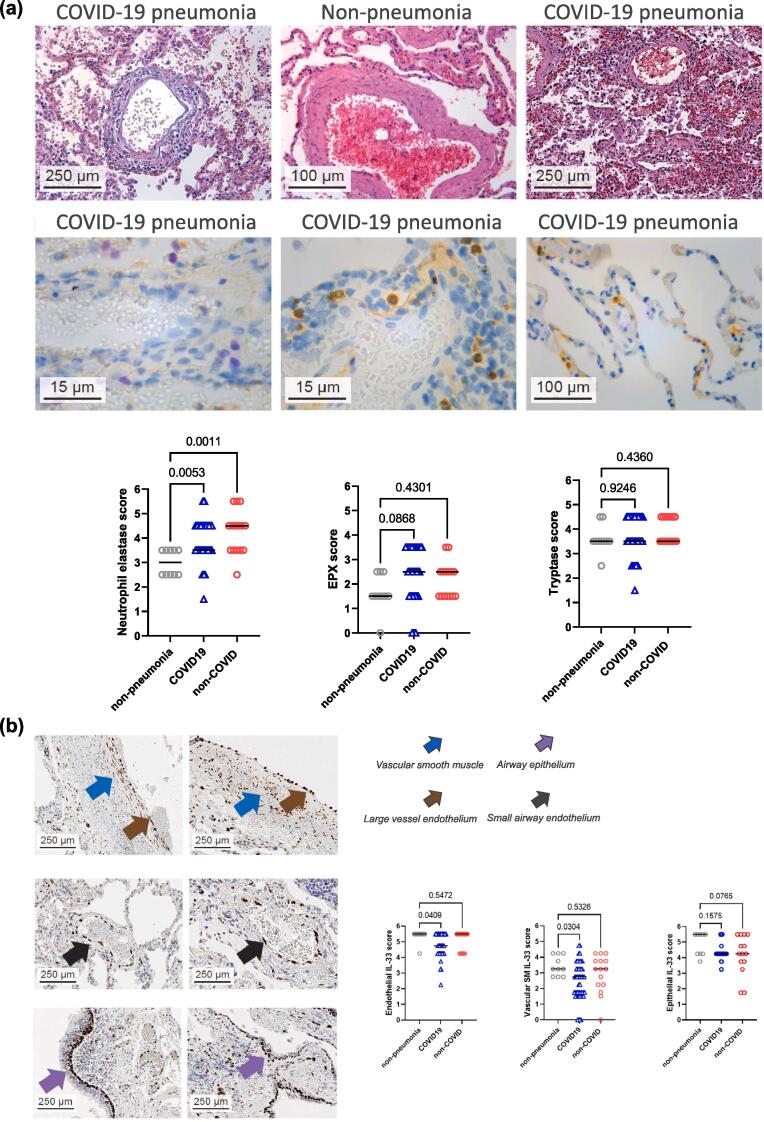

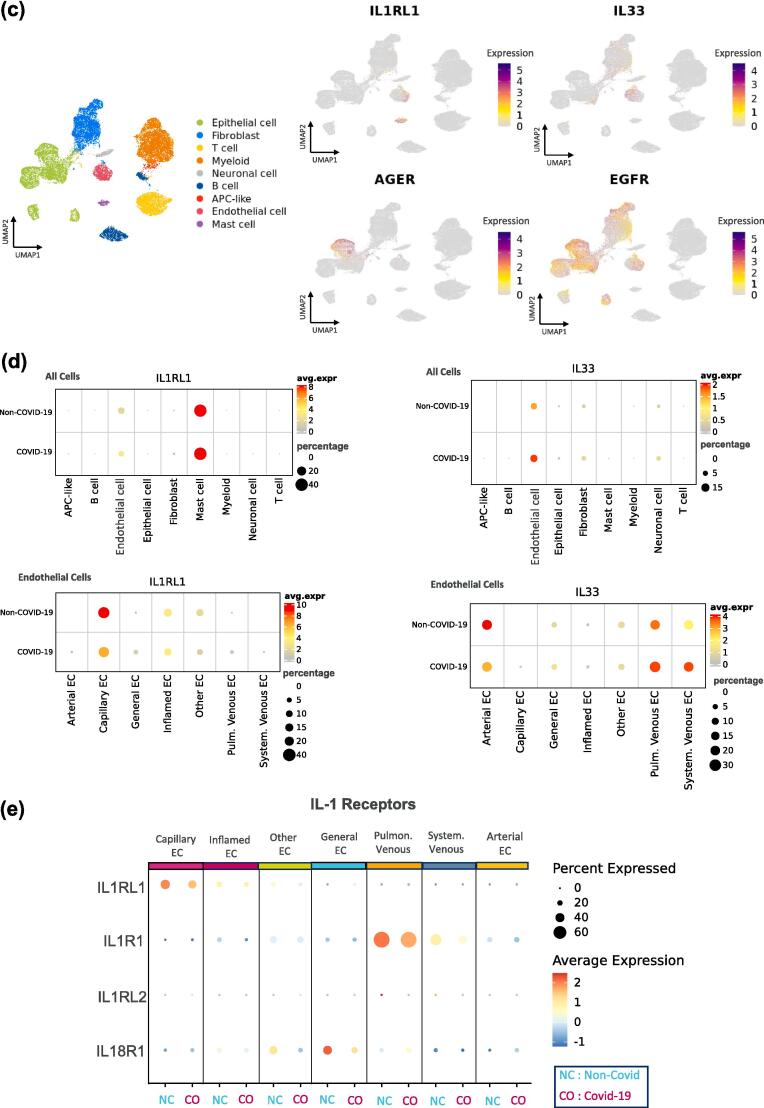
*IL1RL1*, *AGER* and *EGFR* are expressed in alveolar tissues in the lungs from patients with COVID-19. (A) representative images of haematoxylin and eosin-stained tissue slides of lungs from patients with COVID-19 pneumonia. Peri-vascular pathology, including lymphohistiocytic and granulocytic vasculitis in the small arteriole (top left panel). Immune cells were localised in the lumina of vessels in non-pneumonia lungs (top middle panel). Alveolar walls disrupted by microthrombi, loss of pneumocytes and endothelium, abundant fibrin and mixed inflammatory infiltrate (top right panel). Representative images of immunohistochemical-stained COVID-19 pneumonia lung for neutrophil elastase (bottom left panel, yellow stain), EPX (bottom middle panel, brown stain) and mast cell tryptase (bottom right panel, brown stain). Semi-quantitative analysis of immunostaining scores in post-mortem lung tissues from COVID-19 pneumonia (49 samples from 15 patients), non-COVID pneumonia (15 samples from 10 patients) and non-pneumonia lung (10 samples from 5 participants) for neutrophil elastase (left panel), EPX (middle panel), mast cell tryptase (right panel). Median bars and *p* values are shown (one-way ANNOVA tests). (B) representative images of immunohistochemical-stained slides for IL-33 in COVID-19 (left panels) and non-COVID pneumonia (right panels) lungs; large blood vessel (brown arrows, top panels), vascular smooth muscle cells (blue arrows, top panels), small blood vessels (black arrows, middle panels) and basal epithelial cells of bronchi and bronchioles (purple arrows, bottom panels). Semi-quantitative analysis of endothelial IL-33 (left panel), vascular smooth muscle cell IL-33 (middle panel), and epithelial IL-33 (right panel). Median bars and *p* values are shown (one-way ANNOVA tests). (C) UMAP dimensionally reduction plots showing the cell states identified in the complete dataset and the expression levels (log normalised) of *IL1RL1*, *AGER, EGFR* and *IL-33* in post-mortem lungs from patients with COVID-19 and non-COVID participants (11 6313 total cells). (D) dot plots of log-normalised expression levels of *IL1RL1* (left panels) and *IL-33* (right panels) in broad cell types (top panels) and in endothelial cell states (bottom panels) in lungs from patients with COVID-19 (*n* = 19) and patients without COVID-19 (*n* = 7). (E) dot plots of normalised scaled expression profiles of *IL1RL1*, *IL1R1*, *IL18R1* and *IL1RL2* in endothelial cell types in the lungs from patients with COVID-19 and patients without COVID-19. COVID-19 = coronavirus disease 2019; EPX = eosinophil peroxidase; IL = interleukin; UMAP = uniform manifold approximation and projection. (For interpretation of the references to colour in this figure legend, the reader is referred to the web version of this article.)

**Fig. 4:**
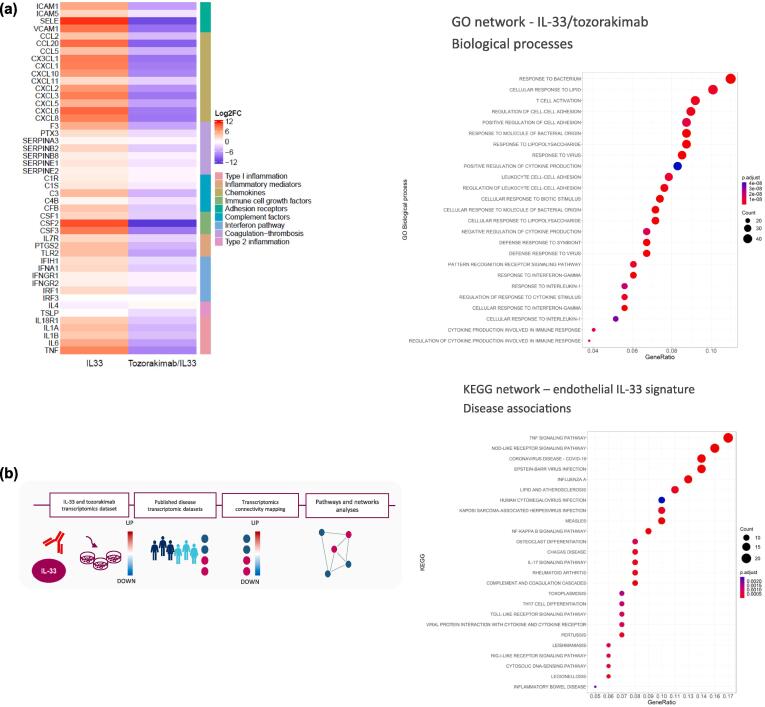

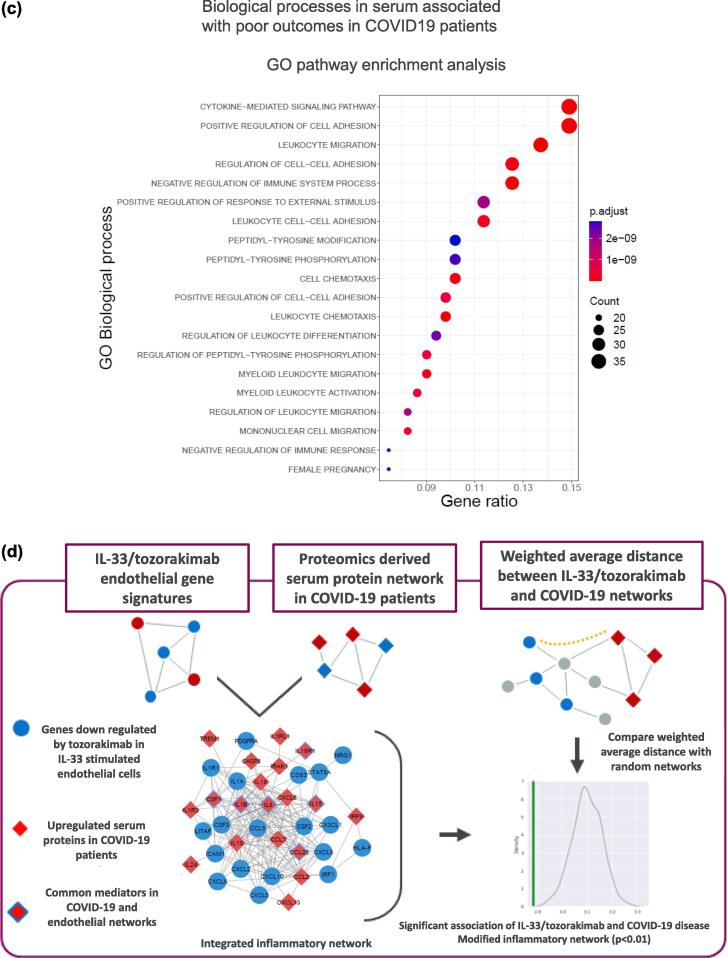
IL-33 drives type I inflammation and coagulation pathways in endothelial cells. (A) heat map (left panel) depicting differential gene expression (log2FC, fdr < 0.05) in HUVECs following treatment for 6 h with IL-33 versus vehicle control (left column) and IL-33/tozorakimab versus IL-33/isotype control (right column). GO pathway enrichment analysis (right panel) number of biological processes for IL-33/tozorakimab-driven gene expression changes (6 h and 24 h, fdr < 0.05 and abs[logFC] > 1). (B) schematic representation of endothelial IL-33 transcriptomic signatures, disease signature mapping and downstream pathways and network analyses (top panel) and KEGG pathway enrichment analysis of disease associations of endothelial IL-33 signatures (bottom panel). (C) GO pathway enrichment analysis of biological processes for serum proteins (*p* < 0.05) associated with poor clinical outcomes in patients hospitalised with COVID-19. (D) integrated omics and network proximity analyses of associations of IL-33/tozorakimab endothelial signatures with serum mediators in COVID-19 patients and quantification of the weighted average distance between serum mediators and endothelial signatures in COVID-19 compared with a randomly distributed network (*p* < 0.01). COVID-19 = coronavirus disease 2019; fdr = false discovery rate; GO = Gene Ontology; KEGG = Kyoto Encyclopaedia of Genes and Genomes; IL = interleukin.

### The IL-33 pathway is associated with poor clinical outcomes in patients with COVID-19

Circulating IL-33/sST2 complex, sST2 and nasal airway IL-33^red^ and IL-33^ox^ significantly associated with the composite clinical endpoint of poor clinical outcomes in patients with COVID-19 (Fig. 2d, Table 1). ^23^ Serum sST2 levels correlated with a greater number of clinical parameters than IL-33/sST2 complex levels (Supplementary Fig. 9). An O-link assay panel analysis of the association between levels of 1094 serum proteins and poor clinical outcomes showed sST2 to be ranked second by adjusted *p* value (Supplementary Table 7), with stronger associations observed with sST2 than for IL-6, IL-1β and tumour necrosis factor-α (TNFα) (Fig. 2e, Table 2). However, because the IL-33/sST2 assay was not included in the O-link proteomics panel, direct comparison of clinical associations of IL-33/sST2 complex with other proteins were not possible. Least absolute shrinkage and selection operator (LASSO) regression identified additional predictive biomarkers, including mediators of inflammation (e.g. semaphorin 4C), oxidative stress (e.g. peroxiredoxin-1), neutrophil activation (e.g. cathepsin C), epithelial remodelling (e.g. hepatocyte growth factor) and endothelial damage (e.g. von Willebrand factor A1, Pentraxin-3); however, soluble RAGE was not associated with poor clinical outcomes (Fig. 2e, Table 2). Serum sST2 levels were more strongly correlated with levels of inflammatory mediators than IL-33/sST2 complex levels (Fig. 2e, Supplementary Fig. 10). In summary, associations between the IL-33 pathway and poor outcomes in patients with COVID-19 indicate a potentially important role of IL-33 in the disease pathology.

**Table 1:** Summary of associations between IL-33 pathway biomarkers and the composite clinical endpoint^#^ in patients with COVID-19.

**Cohort**	**Sample matrix**	**Endpoint**	**Parameter**	**OR (95 % CI)**	***p* value**
Southampton	Serum	Death, admission to an ICU or the need for mechanical ventilation within 30 days of admission	Log(IL-33/sST2 complex [pg/mL])	4.11 (1.71–14.9)	0.004
			Log(Total sST2 [ng/mL])	4.72 (2.40–10.6)	< 0.001
ISARIC4C	Plasma	Death, admission to an ICU or the need for mechanical ventilation within 30 days of admission	Log(IL-33/sST2 complex [pg/mL])	8.21 (3.33–23.0)	< 0.001
	Nasal	Death, admission to an ICU or the need for mechanical ventilation within 30 days of admission	Log(oxidized IL-33 [fg/mL])	1.73 (1.25–2.47)	0.002
			Log(reduced IL-33 [fg/mL])	1.36 (1.14–1.64)	0.001

^#^The composite clinical endpoint was death, admission to an ICU or the need for mechanical ventilation; OR; 95 % CI. CI = confidence interval; COVID = coronavirus; ICU = intensive care unit; IL = interleukin; OR = odds ratio; sST2 = soluble serum stimulation-2.

**Table 2:** Serum proteins associated with poor clinical outcomes in patients with COVID-19.

**Protein**	**OR**	**Adjusted *p* value**
HGF	4.99	0.0040
sST2	3.02	0.0075
SEMA4C	20.93	0.0124
PTX3	11.58	0.0124
IL-6	2.14	0.0124
VWA1	5.74	0.0167
PRDX1	3.24	0.0198
CTSC	17.57	0.0200
IRF9	3.21	0.0248
IL-1β	2.45	0.0463
Soluble RAGE	2.34	0.1605
TNFα	1.54	0.3242

COVID-19 = coronavirus disease 2019; CTSC = cathepsin C; HGF = hepatocyte growth factor; IL = interleukin; IRF9 = interferon-regulatory factor-9; OR = odds ratio; PRDX1 = peroxiredoxin-1; PTX3 = pentraxin 3; RAGE = receptor for advanced glycation end products; SEMA4C = semaphorin 4C; sST2 = soluble serum stimulation-2; TNFα = tumour necrosis factor-α; VWA1 = von Willebrand factor type A1.

### COVID-19 pneumonia is associated with endothelial dysfunction in lung tissue

The histopathology of post-mortem lung tissues from patients with COVID-19 was compared with patients with non-COVID-19 pneumonia and patients who did not have COVID-19 or pneumonia (Supplementary Table 8). It was confirmed that the lungs from patients with COVID-19 had more severe lung pathology than those without COVID-19, including lymphohistiocytic and granulocytic vasculitis, *peri*-vasculitis and microthrombi in the small arterioles and alveolar walls (Fig. 3a). In contrast, immune cells were localised in the lumina of vessels in COVID-negative non-pneumonia lungs (Fig. 3a). The lungs from patients with COVID-19 and those with non-COVID-19 pneumonia contained increased *peri*-vascular immune cell infiltrates, including densely packed neutrophils and low-to-moderate numbers of eosinophils and mast cells (Fig. 3a).

### IL-33 is expressed by epithelial and endothelial barriers in lung tissue

IL-33 expression patterns were examined in lung tissue from patients with COVID-19, patients with non-COVID-19 pneumonia and patients who had neither COVID-19 nor pneumonia. Immunohistochemical staining patterns for IL-33 showed minimal differences between pneumonia and non-pneumonia tissue (Fig. 3b, Supplementary Table 9). IL-33 was expressed in blood vessel ECs (Fig. 3b) and in the basal epithelium of the airways (Fig. 3b), with variable expression in vascular smooth muscle cells (Fig. 3b). Semi-quantitative analysis of staining levels indicated that patients hospitalised with COVID-19 had lower levels of IL-33 expression in the lungs compared with patients without COVID-19 (Supplementary Fig. 3b). IL-33 expression did not correlate with levels of eosinophils, neutrophils, or mast cells (Supplementary Fig. 11a–c). *IL1RL1* gene expression in the lungs was similar in patients with COVID-19 and in those without pneumonia, but expression was upregulated in patients with non-COVID-19 pneumonia (11.9-fold, *p* = 0.0028) (Supplementary Fig. 12a). Gene expression for IL-33^ox^ receptors, *AGER* and *EGFR*, *IL-33* and housekeeping genes in the lungs was similar in all of the subgroups assessed (Supplementary Fig. 12b–g). Overall, *IL-33*, *IL1RL1* and *AGER* expression appears broadly similar in the lungs from patients from all subgroups assessed.

### IL1RL1 is expressed on alveolar capillary ECs associated with gaseous exchange

Published single-cell RNA sequencing datasets were analysed to further investigate patterns of IL-33 receptor expression in the lungs from patients with COVID-19.^27^ *IL1RL1* was expressed on alveolar capillary and inflamed ECs, but not larger vessel EC, and mast cells in lungs from patients with COVID-19 and those without COVID-19 (Fig. 3c and d, Supplementary Fig. 13). In patients with COVID-19, it was confirmed, using cell-specific gene markers (Supplementary Fig. 14), that *IL1RL1* expression was consistent with a population of aerocytes, a specialised type of alveolar capillary EC associated with gaseous exchange and immune cell infiltration.^28^, ^29^ Notably, other IL-1 family receptor genes (*IL1R1, IL18R1, IL1RL2*) were expressed at low or undetectable levels on alveolar capillary ECs (Fig. 3e).^27^, ^30^ *AGER* and *EGFR* were both expressed on type I alveolar epithelial (AT1) cells in the lungs from patients with COVID-19 and in patients without COVID-19 (Fig. 3c, Supplementary Fig. 13). *IL-33* was expressed on airway basal epithelial cells, venous and arterial blood vessels and general ECs, but not on alveolar capillary ECs (Fig. 3c, Supplementary Fig. 14). In summary, gene expression for *IL1RL1*, *AGER*, *EGFR* and *IL-33* were consistent with potential roles in endothelial and epithelial barrier functions in lungs from patients with COVID-19.

### IL-33 drives type I inflammation in endothelial cells

Given the prominent expression of *IL1RL1* (encoding ST2) on endothelial cells, the role of IL-33^red^ in ECs was examined using an *in vitro* cell culture model. Human umbilical vein ECs (HUVECs) were used as a surrogate for lung microvascular ECs (LMVEC) because they have been demonstrated to have similar ST2-pathway and NFkB-dependent inflammatory responses.^18^, ^31^ HUVECs were treated with IL-33^red^ alone or in combination with tozorakimab, an anti-IL-33 monoclonal antibody, that can inhibit both forms of IL-33.^18^ The roles of IL-33 in ECs were examined using an *in vitro* cell culture model. Human umbilical vein ECs (HUVECs) were used as a surrogate for lung microvascular ECs (LMVEC) because they have been demonstrated to have similar ST2-dependent responses.^31^ HUVECs were treated with IL-33^red^ alone or in combination with tozorakimab, an anti-IL-33 monoclonal antibody that can inhibit both forms of IL-33.^18^ Bulk transcriptomics analyses showed 1068 (6 h) and 512 (24 h) genes modified by IL-33^red^ and 1126 (6 h) and 417 (24 h) genes modified by IL-33^red^ and tozorakimab (false-discovery rate of < 0.05 and abs (logFC) > 1) (Supplementary Table 10a–b). Tozorakimab inhibited IL-33^red^ driven gene expression associated with type I inflammation (*IL1α, IL1β, IL6, TNFα, IL18R1*), interferon *(IFNGR1, IFNGR2, IFIH1, IRF1, IRF3, IFNA1, IFI30*), adhesion (*SELE, VCAM1, ICAM1, ICAM5*) and chemokine signalling (*CXCL1, CXCL2, CXCL3, CXCL5, CXCL8, CXCL10, CXCL11, CCL2, CCL5, CCL20, CX3CL1*); coagulation (*SERPINE-1, −2, −B2, −B8, −A3, −F3, PTX3*); and complement factor (*CFB, C1S, C4B, C1R, C3*) (Fig. 4a, Supplementary Fig. 15). IL-33^red^ did not affect expression of type II inflammatory mediators (*IL5, IL13, TSLP, CCL11, CCL24*) (Supplementary Table 10c). Over-represented pathways impacted by IL-33^red^ included response to interferon signalling, host-defence to virus and cell recruitment (Fig. 4a). In summary, this study indicates that IL-33^red^ signalling drives type I inflammatory and coagulation pathways in ECs, consistent with the potential roles of IL-33 in lung endothelial barrier dysfunction in viral LRTD.

### IL-33 signalling associates with signatures associated with broad viral LRTD

To further explore the role of endothelial IL-33 signalling in disease, associations of an IL-33 endothelial signature (adj *p* value < 0.05, Supplementary Table 10b) with 764 disease signatures, from 310 diseases, was evaluated by signature mapping using Indication Discovery Platform (AstraZeneca-DiseaseLinX, previously from Onegevity Health LLC, New York, USA) (Fig. 4b).^32^ The IL-33 signature showed significant associations with respiratory viral disease signatures from *in vitro* models and lung tissue, including COVID-19, influenza A, swine influenza, respiratory syncytial virus, human parainfluenza virus, other infectious diseases and acute lung injury (all p < 0.05; Table 3, Supplementary Table 11). Pathway enrichment analyses (*p* < 0.05) identified additional associations with infectious diseases (Fig. 4b). These results broaden the potential roles for IL-33 in viral LRTD, lung injury and infectious diseases.

**Table 3:** Endothelial IL-33 signatures associate with signatures from a broad range of respiratory viral infections.

**Disease signature**	**Connectivity normScore**	**Adjusted *p* value**	**GSE source**	**Cell source**
Respiratory syncytial virus	−12.41	0	GSE147507	Human A549
SARS-CoV-2 (Calu-3)	−10.05	1.30E-279	GSE147507	Human Calu-3
SARS-CoV-2 (A549-ACE2)	−9.81	1.20E-299	GSE147507	Human A549
Influenza A (IAVDNS1)	−8.74	1.60E-252	GSE147507	Human A549
Human parainfluenza virus-3 (HPIV3)	−8.02	3.70E-177	GSE147507	Human A549
Swine influenza (H1N1)	−7.52	1.50E-192	GSE147507	Human A549
Influenza A (IAVDNS1)	−7.29	1.60E-167	GSE147507	Human A549
Adenovirus infection	−3.60	1.31E-30	GSE4128	Mouse hepatic tissue
Rotavirus infection of children	−3.35	4.15E-25	GSE2729	Human PBMC
West Nile fever	−2.36	1.05E-08	GSE30719	Human retinal pigment epithelial cells
SARS-CoV-2	−2.00	1.40E-03	GSE147507	Human post-mortem lung
Ebola haemorrhagic fever	−1.82	5.49E-04	GSE31747	Human macrophage
Sendai virus infection	−1.72	2.29E-04	GSE10211	Mouse tracheal epithelium
Swine influenza (H1N1)	−1.71	8.46E-04	GSE48466	Human primary lung bronchial epithelial cell
Influenza	−1.70	6.09E-03	GSE3203	B lymphocyte
Influenza A	−1.59	4.33E-03	GSE147507	Ferret nasal wash
Dengue haemorrhagic fever	−1.53	1.09E-02	GSE51808	Human whole blood
Swine influenza (H1N1)	−1.49	1.46E-02	GSE27131	Human peripheral blood

(adjusted *p* < 0.05).

GSE = gene set enrichment; PBMC = peripheral blood mononuclear cells.

### Endothelial IL-33 signature is associated with mediators of poor clinical outcomes in COVID-19

The association between an IL-33 endothelial signature and COVID-19 serum mediators associated with poor clinical outcomes was assessed (Fig. 4c, Supplementary Fig. 16, Supplementary Table 10b). An integrated network of the IL-33 transcriptomic signature and COVID-19 serum protein mediators were in a significantly closer proximity, compared with integration of randomly generated networks of similar size (*p* < 0.01), with a larger connected component and more tightly clustered average shortest path length (Fig. 4d). Overall, results suggest that IL-33-driven EC dysfunction is associated with inflammation in the circulation of patients with COVID-19.

## Discussion

This study describes comprehensive clinical associations of local airway and circulating forms of IL-33 in severe COVID-19. This study provides new mechanistic insights into potential pathological roles of IL-33 in alveolar dysfunction in COVID-19 that is conserved in other common respiratory viral infections that can cause viral LRTD. The development of novel clinical biomarker assays for different IL-33 forms enabled this study to show that increased release of IL-33 from airway epithelial and endothelial barrier cells in patients with COVID-19 was associated with poor clinical outcomes, consistent with roles for these cells in host responses to viral infection.^33^ The results indicate that IL-33 might drive alveolar dysfunction in patients with COVID-19, which could disrupt gaseous exchange and potentially trigger acute respiratory failure (Fig. 5).^34^ Furthermore, these studies demonstrated that IL-33 signalling is also associated with similar mechanisms following other common respiratory viral infections that can cause severe viral LRTD. Of note, in a phase 2 study (ACCORD-2), therapeutic targeting of IL-33 with tozorakimab demonstrated some clinical benefit in patients with severe COVID-19, with enhanced efficacy observed in patients with high serum levels of IL-33/sST2 complex.^35^ Although the ACCORD-2 study failed to meet its primary endpoint, time to clinical response, results warranted further investigation to see if targeting IL-33 can reduce hypoxia and improve outcomes in a broad population of patients with severe viral LRTD (TILIA; NCT05624450).^36^

Two independent cohorts of patients hospitalised with COVID-19 from the pre-vaccination period were utilised, that likely to reflect responses to pre-alpha or alpha strains of SARS-CoV2. The Southampton cohort had marginally higher levels of mortality and a marginally greater number of patients who reached the composite clinical endpoint^23^ compared with the ISARIC4C cohort. Lung sampling was not possible in these patients; therefore, nasal sampling was employed to assess airway biomarker levels.^37^ Levels of IL-33^ox^, but not IL-33^red^ (which has a short half-life^16^), were significantly increased in nasal samples from patients with COVID-19 compared with healthy participants. However, nasal levels of both IL-33^red^ and IL-33^ox^ were both associated with poor clinical outcomes. This apparent discrepancy is unexplained but is consistent with the hypothesis that the release of IL-33^red^ is increased in patients with COVID-19, compared with healthy participants, but is more rapidly oxidised in patients with COVID-19. Indeed, tissue inflammation is associated with an enhanced oxidative extracellular environment.^38^, ^39^ Overall results indicate that the nasal mucosa might be a surrogate for the lower airway to aid identifying biomarkers that are predictive of poor clinical outcomes. The associations with poor clinical outcomes were stronger with circulating IL-33/sST2 complex and sST2 levels than with IL-6, IL-1β and TNFα, which were proposed as targets for treatment of COVID-19.^40^ Levels of IL-33 redox forms and levels of sST2 were poorly correlated, potentially owing to differences in regulation of expression and release of these biomarkers.^41^ However, sST2 levels correlated with levels of other inflammatory mediators; this is consistent with evidence showing that other inflammatory mediators beyond IL-33 can increase sST2 expression^42^ and that tozorakimab does not inhibit sST2 levels.^35^, ^43^ Overall, association of the IL-33 pathway biomarkers with poor outcomes in patients with COVID-19 indicates the potential to develop diagnostics to stratify patients and identify who might most benefit from therapeutics targeting IL-33.^35^

In this study, it was observed that COVID-19 pneumonia, and to lesser extent, non-COVID-19 pneumonia lung tissue had airway and alveolar endothelial pathology. Here, and other reports, have shown associations of endothelial biomarkers with poor clinical outcomes in COVID-19 patients.^34^ IL-33 was localised in airway epithelial and endothelial barriers in the lungs from patients with and without pneumonia, similar to other diseases.^17^ Consistent with release of pre-stored IL-33 following viral infection, lower levels of IL-33 staining in COVID-19 lungs was observed with no change in *IL-33* gene expression. These results contrast with a study reporting complete depletion of IL-33 in the lungs from patients with COVID-19,^44^ which might be explained by differences in tissue preparation. Notably, single-cell transcriptomic analysis showed for the first time that *IL-33* was expressed in airway blood vessel ECs, whereas *IL1RL1* (but not other IL-1 family receptors) was expressed by aerocytes.^27^, ^45^ Expression of *IL1RL1* in aerocytes from healthy participants and patients with COPD has been reported. 28, 29Therefore, although IL-33 and IL-1 can drive similar responses in ECs,^46^ the previously unappreciated relative expression patterns indicate that ST2 signalling might have differentiated roles in aerocyte dysfunction and impairment of gaseous exchange.^45^ Expression of *IL1RL1* in lung mast cells in patients with COVID-19 suggests that IL-33 signalling in these cells might contribute to alveolar dysfunction.^47^ *IL1RL1* expression was not detected in other immune cells, possibly owing to the transient changes in *IL1RL1* expression associated with viral infection.^48^ Here we report for the first time that *AGER* and *EGFR* were both expressed on AT1 cells from patients with COVID-19, indicating that IL-33^ox^ might drive increased alveolar epithelial remodelling and mucus hyper-secretion in viral LRTD.^49^

Lung endothelial dysfunction is associated with viral LRTD, asthma and COPD pathology.^34^, ^50^, ^51^ HUVECs were used as a surrogate *in vitro* model for lung endothelial cells in this study.^31^ Lung endothelial cells and HUVECs expressed *IL1RL1* and IL-33 robustly increased ST2-dependent inflammatory responses^18^, ^31^ In contrast, *AGER* was not detected on lung endothelial cells from healthy participants or patients with COVID-19 in this study. RNA sequencing of HUVECs showed IL-33^red^ can potentially drive type I inflammation, neutrophil and other immune cell infiltration, interferon signalling and coagulation that substantially extend understanding compared with related studies in LMVEC and other endothelial cells.^18^, ^31^ IL-33-driven interferon signalling can contribute to airway barrier dysfunction, neutrophilic inflammation^52^ which may contribute to poor patient outcomes. Furthermore, IL-33 increased endothelial gene expression of chemokines, including *CXCL10* and *CCL5,* that can increase recruitment of natural killer and CD8^+^ T-cells, which have potential pathological roles during viral exacerbations.^11^, ^48^, ^53^ Of note, IL-33 can also enhance interferon-γ release from peripheral blood mononuclear and natural killer cells.^18^ IL-33 increased endothelial gene expression for tissue factor and serpins consistent with promoting coagulation observed in the lungs from of patients with COVID-19.^54^ IL-33 released from platelets and red blood cells might also contribute to endothelial dysfunction and coagulation in viral LRTD.^55^, ^56^ In summary, the results provide mechanistic insights for roles of the IL-33^red^/ST2 pathway on ECs in viral LRTD and respiratory exacerbations that are associated with type I and neutrophilic inflammation,^11^, ^57^ and steroid resistance.^58^, ^59^ Furthermore, disease signature mapping provides additional insights for the targeting of IL-33-associated diseases, including pneumonia, pulmonary injury and infection.^60^, ^61^, ^62^

A novel panel of immunoassays was developed and extensively characterised for this study to selectively detect IL-33^red^, IL-33^ox^ and IL-33/sST2 complex in clinical samples. Commercial IL-33 assays have proven unreliable for clinical use because of poor selectivity and sensitivity, ^20^ and new assays have only been identified for IL-33^red^ but not IL-33/sST2 complex or IL-33^ox^.^26^, ^63^ This study indicates that released IL-33 is active in local tissues but is rapidly inactivated in the circulation owing to high sST2 levels. Previously, measurements of sST2 with commercial assays have been inconsistent, potentially due to differential detection of IL-33/sST2 complex.^20^ This study showed that the Presage assay detects sST2 independent of complexing with IL-33. Overall, here we report a major advance in the development of a reliable panel of ultra-sensitive biomarker assays for IL-33 and demonstrate their utility for clinical associations in this study and for target engagement and precision medicine studies.^35^, ^43^

This study has several limitations. Access to longitudinal patient samples were not available for this study to compare the trajectory of IL-33 release with sST2. Previously, it has been reported that sST2 levels remain high for 30–50 days in patients hospitalised with COVID-19 with poor outcomes.^64^ Additionally, given the associations between the IL-33 pathway and viral LRTD, it will be interesting to compare biomarker associations and disease mechanisms in patients with COVID-19 and with other viral LRTDs. It should be noted that comparisons of biomarker and lung tissue studies should be interpreted cautiously because results were derived from different patient cohorts. It is important to acknowledge that single-cell transcriptomic analyses have limitations for the detection of low-abundance transcripts (e.g. *IL1RL1*), particularly in rare and fragile cell types, including granulocytes. Furthermore, the lack of validated tools precluded analysis of the localisation of ST2 or *IL1RL1* in lung tissue. HUVECs, derived from a single donor, were used as a surrogate *in vitro* model for investigating potential roles for IL-33 in the lung endothelium. Because *AGER* and *EGFR* were both expressed in the alveolar epithelium in patients with COVID-19, further investigation is warranted to understand if IL-33^ox^-RAGE-EGFR signalling pathway in the alveolar epithelium contributes to lung remodelling following respiratory viral infections.^49^ It is noteworthy that *AGER* and *EGFR* have been localised in COPD lung airways and IL-33^ox^ signalling can drive mucus hypersecretion in small and large airway air–liquid interface cultures.^19^ Although *AGER* was not detected on lung ECs by single cell transcriptomics the biology of IL-33^ox^ on endothelial cells is potentially worthy of further research. IL-33 appears to have important roles in both innate and adaptive immunity during viral host defence^5^, ^6^, ^7^ and viral LRTD;^9^, ^10^ consequently, it is important to understand the mechanisms (e.g. levels of immune cells in the airway)^65^ influencing host protective and pathological roles following viral infection.

This study has demonstrated associations between IL-33 and poor outcomes in patients hospitalised with COVID-19 and mechanistic insights consistent with roles for IL-33 in viral LRTD. Although a causal relationship cannot be inferred from this study, these results provide a rationale to evaluate potential pathological roles of IL-33 in patients with viral LRTD. Therapeutic targeting of IL-33 has shown some clinical benefits in respiratory diseases,^2^, ^3^ potentially including COVID-19.^35^ However, larger trials are required to understand the potential clinical benefit in patients with viral LRTD at risk of acute respiratory failure.^36^

## Materials and methods

Study samples and experimental methods used are summarised in Fig. 1. Detailed methods are described in the online supplement.

### University of Southampton cohort

The study was approved by the South Central – Hampshire A Research Ethics Committee (20/SC/0138) and comprised a cohort of patients hospitalised with COVID-19.^66^ Severe acute respiratory syndrome coronavirus 2 (SARS-CoV-2) infection was confirmed using QIAstat-Dx Respiratory panels (Qiagen, Manchester, UK). Blood samples were collected by venesection in separating tubes and clotted before serum collection by centrifugation. Demographic and clinical data were collected at enrolment and outcomes were assessed retrospectively. Patient health status was recorded using the National Early Warning Score 2. Laboratory tests and treatment data were collected using Real-time Analytics for Clinical Trials platform (Experimental Cancer Medicine Team, Manchester, UK).^23^

### International Severe Acute Respiratory Infection Consortium Coronavirus Clinical Characterisation Consortium (ISARIC4C) cohort

Ethical approval was provided by the South-Central Oxford Ethics Committee (13/SC/0149), Scotland A (20/SS/0028) Ethics Committee and World Health Organization (WHO) Research Ethics Review Committee (RPC571 and RPC572). Clinical data and samples were collected from 182 patients who were hospitalised with polymerase chain reaction-confirmed SARS-CoV-2 infection using the ISARIC4C/WHO Clinical Characterisation Protocol for Severe Emerging Infections.^67^ Nasal mucosal lining fluids (MLFs) were collected by nasosorption (Nasosorption FX-i, Mucosal Diagnostics, Midhurst, UK) and eluted in phosphate-buffered saline containing 0.05 % Tween 20 (volume per volume [v/v]), 0.1 % bovine serum albumin (weight per volume [w/v]) and 1 % (v/v) Triton X-100.^37^ Nasal MLF samples and ethylenediaminetetraacetic acid-plasma were stored at −80 °C and were used for a maximum of two freeze–thaw cycles.

### Healthy participants

Demographics and clinical procedures for healthy participants (NCT03096795) were reported previously.^43^ Healthy participants had no symptoms of infection and clinical samples were collected in the pre-COVID-19 era.

### Post-mortem lung tissue samples

Participants were volunteers in the Arizona Study of Aging and Neurodegenerative Disorders (AZSAND), a longitudinal clinicopathological study of aging, cognition and movement. Autopsies were performed by the Banner Sun Health Research Institute Brain and Body Donation Program, Sun City, AZ, USA.^68^ All participants signed an institutional review board-approved informed consent form for autopsy and post-mortem tissue donation. Post-mortem lung tissue samples were used in accordance with the Declaration of Helsinki and in compliance with national and local regulatory guidelines (Study #1132516, IRB #20120821). Medically licensed pathologists performed all diagnostic examinations using methods standardised by the AZSAND, which consisted of gross and microscopic examination, including pathologist assessment of both brains and peripheral organs. All *in vivo* clinical diagnoses were determined by licensed medical laboratories using US Food and Drug Administration Emergency Use Authorization protocols. Use of post-mortem lung tissue for histopathology, immunohistochemistry and RNA sequencing analyses is described in the supplementary methods.

### Commercial IL-33 immunoassays

Pro Th17 (Bio-Plex), Duoset ELISA (Biotechnie) and U-plex (Mesoscale Discovery (MSD)) IL-33 immunoassays were assessed using recombinant IL-33 protein standards.^16^, ^18^, ^19^.

### Development of IL-33^red^, IL-33^ox^ and IL-33/sST2 complex assays

Ultra-sensitive electrochemiluminescent immunoassays for IL-33^red^, IL-33^ox^ and IL-33/sST2 complex were developed on the S-plex platform (MSD).^69^ Recombinant protein standards, IL-33^red^, IL-33^ox^ and sST2-Flag-His, were generated as described previously.^16^, ^18^, ^19^ IL-33/sST2 complex was prepared by incubating IL-33^red^ and sST2 (molar ratio 1:10) for 30 min and stored at −80C. mAbs were identified by hybridoma, HTRF IL-33 binding assays and DNA sequencing (Supplementary Table 3). Detection antibodies were SULFO-TAG labelled (MSD). Optimal capture and detection antibody pairs were identified through comprehensive screening of 11 anti-IL-33^red^ and 3 anti-IL-33^ox^ monoclonal antibodies derived from hybridoma, 5 commercial anti-IL-33 antibodies (Enzo Life Sciences and Adipogen), and 4 anti-ST2 antibodies (R&D systems) (Supplementary Table 3) in microtitre plate format using recombinant IL-33^red^, IL-33^ox^ and IL-33/sST2 complex and human serum from healthy participants. IL-33/sST2 complexes were detected using an anti-IL-33^red^ capture and an anti-ST2 detection mAb. Optimal antibody pairs, antibody concentrations and diluents were selected based on highest signal to noise ratio and lowest LLOD in assay standard curves. Spike and recovery, dilution linearity and parallelism were performed in serum, EDTA- and heparin-plasma and nasal mucosal lining fluids from *n* = 5 individuals. Assay selectivity was confirmed as < 0.5 % non-specific signals using recombinant human IL-1α, IL-1β, IL-18, IL-1RA, IL-36γ, IL-25, IL-5, IL-6, IL-8, RAGE, TSLP (R&D systems). Tolerance of assays to common endogenous substances were confirmed in serum and plasma (2 mg/mL haemoglobin, 0.004 mg/mL conjugated- and 0.15 mg/mL unconjugated-bilirubin, and 2.5 mg/mL triglyceride-rich lipoproteins). Acid-treatment of clinical samples did not increase signals in our assays in contrast to other reports.^63^

### IL-33^red^ and IL-33^ox^ immunoassays

Plates were washed (3x PBS/0.05 % (v/v) Tween-20), coated with capture Ab (0.25 µg/mL) and incubated for 1 h, washed, then protein standards (recombinant IL-33^red^ or IL-33^ox^) or samples incubated for 2 h with TURBO-boost labelled detection Ab 0.025 µg/mL (IL-33^red^ assay) or 0.08 µg/mL (IL-33^ox^ assay).

### IL-33/sST2 complex immunoassay

Plates were washed, coated with capture Ab (0.25 µg/mL), and incubated for 1 h, washed, then protein standard (recombinant IL-33/sST2 complex) or samples incubated for 1.5 h with blocking buffer and washed. Plates were incubated for 1 h in TURBO-boost labelled detection Ab (0.1 µg/mL). All assay plates were washed, incubated for 1 h in S-PLEX detection solution (MSD), washed and incubated in GOLD Read Buffer A (MSD) and then read on a MESO SECTOR S600 instrument.

### sST2 immunoassay

sST2 was measured in patient samples using the Presage assay (Critical Diagnostics) according to manufacturer’s instructions.

## Author guidelines

All authors were involved in the drafting of the manuscript, provided critical revisions for important intellectual content, approved the final version submitted for publication and agreed to be accountable for all aspects of the work.

## Data sharing

Data underlying the findings described in this article may be obtained in accordance with AstraZeneca’s data sharing policy described at https://astrazenecagrouptrials.pharmacm.com/ST/Submission/Disclosure.

## CRediT authorship contribution statement

ICS, NvZ, JAC, VAN, KT, HK, ZL, CMcC, DGR, EE, MAG, KH, DMcC, AF, DS, AF, ESC, RT, ZB, AP, PJMO, MGS, JKB and TW were responsible for writing the original manuscript draft and reviewing and editing the manuscript.

## Funding

AstraZeneca part-funded this study and participated in the study design, data collection, data analysis and data interpretation. AstraZeneca reviewed the publication, without influencing the opinions of the authors, to ensure medical and scientific accuracy and the protection of intellectual property. The corresponding author had access to all data in the study and had the final responsibility to submit the manuscript for publication. This work is supported by grants from: the UK National Institute for Health and Care Research (NIHR; award CO-CIN-01); the UK Medical Research Council (MRC; grant MC_PC_19059); the NIHR Health Protection Research Unit (HPRU) in Emerging and Zoonotic Infections at the University of Liverpool in partnership with Public Health England (now UK Health Security Agency [UKHSA]), in collaboration with Liverpool School of Tropical Medicine, Liverpool, UK; Wellcome Trust and Department for International Development (DID; 215091/Z/18/Z); the Bill & Melinda Gates Foundation (OPP1209135), the University of Oxford, Oxford, UK (NIHR award 200907); and Liverpool Experimental Cancer Medicine Centre, Liverpool, UK, for infrastructure support for this research (grant reference C18616/A25153); Wellcome Trust Senior Research Fellowship (JKB; 223164/Z/21/Z), UKRI grants MR/Y030877/1, MC_PC_20004, MC_PC_19025, MC_PC_1905, MRNO2995X/1 and MC_PC_20029; Sepsis Research (Fiona Elizabeth Agnew Trust) and a BBSRC Institute Strategic Programme Grant to the Roslin Institute (BB/P013732/1, BB/P013759/1); Baillie Gifford and the Baillie Gifford Science Pandemic Hub at the University of Edinburgh; the NIHR (award CO-CIN-01); the UK Medical Research Council (MRC; grant MC_PC_19059); the NIHR Health Protection Research Unit in Emerging and Zoonotic Infections at the University of Liverpool in partnership with Public Health England (now the UKHSA). The views expressed in this paper are those of the authors and not necessarily those of the Department of Health and Social Care, NIHR, MRC, Wellcome Trust, or UKHSA.

## Declarations of competing interests

ICS, NVZ, VAN, KT, HK, ZL, CM, DGR, EE, MG, KH, ESC, ZB, DS, AF and AP are employees of AstraZeneca and may hold stock or stock options in AstraZeneca. JAC is a former employee of AstraZeneca and may hold stock or stock options in AstraZeneca. PJMO has received fees for scientific advisory boards from GSK, Moderna, Seqirus, Janssen and Sanofi Pasteur. MGS has received grants from the Department of Health and Social Care National Institute for Heath and Care Research, MRC, HPRU in Emerging and Zoonotic Infections, and the University of Liverpool during the conduct of the study; and has received other grants from Integrum Scientific LLC and Greensboro outside the submitted work. TW has received grants and fees from AstraZeneca, Bergenbio, Boehringer Ingelheim, Chiesi, GSK, Janssen, Olam, MMH, Synairgen, Union Chimique Belge and Valneva. DM, RT and JKB have no conflict of interests.
