# Supplementary material for: IL-33 is associated with alveolar dysfunction in patients with viral lower respiratory tract disease
Source: Mucosal Immunol. 2025 Apr;18(2):312–25. doi: 10.1016/j.mucimm.2024.12.001 (PMC11982439; doi:10.1016/j.mucimm.2024.12.001)
Supplement: Supplementary Data 1 [file mmc1.pdf]

Supplementary material and methods

Statistical analyses for biomarker studies

We analysed the association of IL33/sST2 complex and sST2 with 46 clinical parameters in 100 hospitalised patients with COVID-19. A composite end point for severe COVID-19 disease was defined as admission to the ICU and/or need for mechanical ventilation and/or death within 30 days of hospitalisation.[1] For the purpose of analysis, measurements of biomarkers were log transformed in R.[2] Association of biomarkers with continuous clinical parameters was performed using a linear regression with discrete outcomes using a logistic regression in R.[2] The models were adjusted for age, gender, and National Early Warning Score-2.[3] To identify biomarkers that were independent predictors of poor clinical outcomes in COVID-19 patients, we performed a Least absolute shrinkage and selection operator (LASSO) regression with L1 regularisation using the glmnet package in R statistics. As the sample size was only 100 participants, we did not split the data into test and training but used generalised cross validation to determine the optimal value of lambda for predictions. Cross validation was performed 1000 times and the average of statistics was used to derive effect estimates and *p* values for the different features selected.

Post-mortem lung tissue for single nucleus RNA sequencing studies

Clinical procedures and data associated with post-mortem lungs from COVID-19 (n = 19) and non-COVID patients (n = 7) for single cell RNA sequencing analyses were published.[4]

Anti-IL-33 antibody isolation and characterisation is summarised in Supplementary Fig. 2a.

Immunisation for anti-IL33 antibodies

Female CD1 mice (6-8 weeks of age; Charles River Laboratories) were injected subcutaneously with 20 µg recombinant human IL-33^{red} in Freund's complete adjuvant (Sigma-Aldrich). Three subsequent boosts of 20 µg recombinant IL-33^{red} in Freund's incomplete adjuvant (Sigma-Aldrich) were administered through subcutaneous injection 7, 14 and 22 days after the initial prime. Two days after the fourth boost, a final injection of 20 µg recombinant IL-33^{red} in phosphate-buffered saline (PBS) was administered intraperitoneally. Four days later the mice were sacrificed, and lymph nodes harvested. All work was conducted to UK Home Office ethical and husbandry standards under the authority of an appropriate project license.

Hybridoma generation and culture

Cells were isolated from lymph nodes by mechanical disruption using the gentleMACS dissociator (Miltenyi) and subsequently fused with SP2/0 myeloma cells (American Type Culture Collection, ATCC) using a Legacy ECM2001 electrofuser (BTX Molecular Delivery Systems). Fused cells were resuspended in semi-solid selection media (CloneMatrix concentrate; Genetix) supplemented with FITC conjugated anti-mouse IgG polyclonal antibody (Jackson Immunolabs), DMEM powder (Gibco), 20% (v/v) FCS (SAFC Biosciences), 2% (v/v) glutaMAX (Gibco), 1% (w/v) sodium pyruvate (Sigma-Aldrich), 1% (v/v) penicillin/streptomycin (Gibco), 10% (v/v) hybridoma cloning factor (Roche), 2% (v/v) oxaloacetate/pyruvate/insulin (Sigma-Aldrich), 2% (v/v) hypoxanthine/azaserine (Sigma-Aldrich). The cells were divided into omni trays (Nunc) and cells grown at 37°C in a 7.5% CO_2-enriched atmosphere. 13-16 days post-fusion IgG secreting hybridomas were detected and picked into 96 well plates (Costar) containing media [DMEM, 20% (v/v) FCS, 2% (v/v) glutaMAX, 1% (v/v) penicillin/streptomycin, 10% (v/v) hybridoma cloning factor, 2% (v/v) oxaloacetate/pyruvate/insulin, 2% (v/v) hypoxanthine and thymidine (Sigma-Aldrich)] using the ClonePix FL colony picker (Molecular Devices). Hybridomas were grown for 3-7 days prior

to supernatant to harvest using a MiniTrak robotic liquid dispenser (Perkin Elmer). Example serum anti-IL-33 titres are shown in Supplementary Fig. 2b.

cDNA preparation and antibody variable chain sequencing

Hybridoma mRNA for heavy and light chain antibody variable regions were isolated with Oligo $(dT)_{25}$ magnetic beads (Novagen) on a Kingfisher 96 automated magnetic separator (Thermo Fisher Scientific). Contaminating SP2/0 MOPC abVκ mRNA were removed by targeted digestion with RNaseH (NEB) at 37C for 1 hour. Purified mRNA were transcribed to cDNA using superscript III reverse transcriptase (Invitrogen) at 44C for 1 hour and tailed with poly(G) by incubation with excess dGTP and terminal transferase (New England Biolabs) at 37C for 1 hour. Variable light and heavy chain genes were amplified using Taq polymerase (Thermo Fisher Scientific) in separate reactions using oligo$(dC)_{25}$ and primers specific to either the CH1 or kappa constant domain regions. Chain termination method was used to sequence the PCR product.

Hybridoma IgG purification

Selected hybridomas were cultured in serum free media [HL-1(Lonza), 2% (v/v) HybER-Zero (Statins Serum Institute), 2% (v/v) glutaMAX (Gibco)] for 10 days at 37°C in a 7.5% CO_2-enriched atmosphere. IgG was purified from the culture supernatants using ProPlus resin bed Phytips (Phynexus) on the MiniTrak robotic liquid dispenser. Absorbance of purified material was determined using an EnVision® plate reader (Perkin Elmer) at 280 nm and IgG concentration calculated using mouse IgG isotype calibration curves.

Recombinant mouse IgG expression and purification

Clones of interest were converted to mouse IgG1 antibody format[5] with the following modifications. An OriP fragment was included in the expression vectors to facilitate use with CHO-transient cells and to allow episomal replication. The VH domain was cloned into a vector containing the mouse heavy chain constant domains and regulatory elements to express

whole IgG1 heavy chain in mammalian cells. Similarly, the VL domain was cloned into a vector for the expression of the mouse light chain (kappa) constant domains and regulatory elements to express whole IgG light chain in mammalian cells. To obtain IgGs, the heavy and light chain IgG expressing vectors were transfected into CHO-transient mammalian cells[6], and IgGs were expressed and secreted into the medium. Harvested media were filtered prior to IgG purification from culture supernatant using an AKTAxpress (GE healthcare) and 1 mL MabSelect Sure columns (GE healthcare). IgGs were eluted from the columns using 0.1 M Sodium Citrate (pH 3.0) and buffer exchanged into PBS using PD10 columns (GE Healthcare). Antibody concentration was determined spectrophotometrically using extinction coefficients based upon the individual amino acid sequences of the IgGs.[7] Integrity and purity of purified IgGs was determined including SDS-PAGE and HPLC-SEC.

HTRF binding assays

Biochemical assays using Homogeneous Time Resolved Fluorescence resonance energy transfer (HTRF) technology were established to measure the binding of test samples to IL-33^{red}, IL-33^{ox} or IL-33/sST2 complex (Supplementary Fig. 2c). Samples were incubated with 10 nM biotinylated IL-33^{red} pre-mixed with 10 nM XL665 conjugated anti-mouse Fc IgG (PerkinElmer, Cisbio) and 1.7 nM europium cryptate conjugated streptavidin (PerkinElmer, Cisbio) or 10 nM biotinylated IL-33^{ox} pre-mixed with 10 nM XL665 conjugated anti-mouse Fc IgG (PerkinElmer, Cisbio) and 1.7 nM europium cryptate conjugated streptavidin (PerkinElmer, Cisbio) or 5 nM IL-33^{red} (Viva) pre-mixed with a 1 in 2000 dilution of europium cryptate conjugated sST2 and 10 nM XL665 conjugated anti-mouse Fc IgG (PerkinElmer, Cisbio) for 4 hours at room temperature followed by 15 hours at 4C. All reagents were prepared in a buffer containing phosphate-buffered saline (Gibco), 0.4 M potassium fluoride (VWR) and 0.1% (w/v) bovine serum albumin (Sigma). The fluorescence was measured on an EnVision plate reader (PerkinElmer) using an excitation 320 nm filter with the emission filters 665 nm and 590 nm. The raw data was initially analysed using the equation 665 nm / 590 nm * 10,000 and expressed as % DELTA F using the equation (sample ratio - negative

control ratio / negative control ratio *100). HTRF data for selected monoclonal antibodies (mAbs) used for finalised assays are shown in Supplementary Fig. 2d.

Affinity measurements

Prior to the SPR analysis monomeric fractions of recombinant IL-33^{red}, recombinant sST2 or IL-33/sST2 complexes[8-10] were isolated by Size exclusion chromatography (SEC) using Superdex 200 increase P/N 28-9909-44 equilibrated with Dulbecco's PBS. Affinities and kinetics for the IgG panel interactions with IL-33^{red}, IL-33^{ox} and IL-33/sST2 complexes were measured by surface plasmon resonance on an 8K Biacore chip (Cytiva Ltd). Briefly antibodies were directly amine coupled to C1 chips using materials and reagents from Cytiva Ltd. Antibody Interactions with antigen dilutions (IL-33 forms or IL-33/ST2 complexes) were performed at 25°C and a flow rate of 50 μL min^{-1} using appropriate dilutions of antigen within the Single-Cycle Kinetics method with the dissociation measured for 10 minutes. Affinities of anti-IL-33 capture mAbs (Supplementary Table 4) were broadly consistent with limits of detection in immunoassays (Supplementary Table 6).

Proteomics

Relative expression levels of 1094 protein biomarkers in serum samples from 100 hospitalised patients with COVID-19 (Southampton cohort) using PEA technology (O-link proteomics) and target96 assays panels (inflammation, immune regulation, oncology II and III, metabolism, organ damage, neuro-exploratory, neurology, development, cardiovascular II and III). Measures were removed if there was a QC flag warning generated by O-link or if measures were below the limit of detection. We analysed the association of individual proteins with the severe COVID endpoints whilst correcting for age, gender and ethnicity in a logistic regression using R {R Core Team (2021).[2] To account for multiple testing a False Discovery rate of 0.1 was applied to the data.

Post-mortem lung tissue histopathology

Pathological examinations were performed by medically licensed pathologists using standard Arizona Study of Aging and Neurodegenerative Disorders (AZSAND) methods and consisted of gross and microscopic examination. Formalin-fixed and paraffin-embedded (FFPE) tissues (Banner Sun Health Research Institute, Sun City, AZ, USA) were stained with haematoxylin and eosin (H&E) for pathological assessment of organs including bilateral upper and lower lobe lung samples to assess histopathology consistent with COVID-19 pneumonia. Clinical diagnoses were rendered by licensed medical laboratories using US Food and Drug Administration (FDA) Emergency Use Authorization (EUA) protocols. Post-mortem nasopharyngeal swabs (BD# 220531) from all BSHRI cases were assayed for SARS-CoV-2, Hepatitis B and C and HIV RNA using FDA EUA protocols at Clinical Laboratory Improvement Amendments (CLIA)-approved laboratories (Sonora Quest, a division of Quest Diagnostics and Stanford Health Care in Stanford, California, USA). COVID-19 pneumonia samples were confirmed positive for SARS-CoV-2 RNA and by pathological examination whereas non-COVID-19 pneumonia and non-pneumonia samples were confirmed negative for SARS-CoV-2 RNA. All samples were negative for Hepatitis B, -C and HIV. Patient samples, demographics, clinical and pathology reports associated with participants samples are summarised in Supplementary Table 8.

Lung tissue immunohistochemistry

Immunohistochemical (IHC) detection of IL-33 protein was performed on FFPE post-mortem lung tissue samples (Banner Sun Health) from 15 patients with COVID-19 pneumonia, 10 patients with non–COVID-19 pneumonia, and 5 patients without pneumonia or COVID-19. A total of 74 FFPE bilateral upper and lower lobe lung samples lung samples (49 samples from patients with COVID-19 pneumonia, 15 from patients with non–COVID-19 pneumonia, and 10 from patients without pneumonia) were provided as unstained, 4-µm sections mounted on positively charged, microscope slides. For IL-33 mono-plex chromogenic IHC, FFPE sections were deparaffinised and stained with a Leica Bond RX IHC/ISH research slide staining system

(Leica Microsystems) including a heat-induced epitope retrieval pre-treatment step using ER2 (Leica Microsystems), and endogenous peroxidase was blocked using peroxidase for 8 min, and fish skin gelatin for 15 min. Sections were incubated with an anti-human-IL-33 antibody (0.85 μg/mL Nessy-1, #GTX14709, GeneTex) for 25 min at 37ºC. The IL-33 primary antibody was detected using secondary anti-mouse polymer conjugated to HRP (Mouse Refine Detection Kit, Leica Microsystems), followed by diaminobenzidine chromogen substrate (DAB Refine Detection Kit, Leica Microsystems) incubation for 10 min. All slides were counterstained with hematoxylin. Non-immune mouse isotype antibody (IgG) was included as a negative control (Mouse isotype IgG1, # mAb002, R&D Systems). All slides were scanned at 20–40X magnification using an Aperio AT2 (Leica Biosystems) digital slide scanner and examined by a board-certified pathologist using ImageScope v12.4.3.7001 (Leica Microsystems). Descriptive findings were recorded via text and digital photomicroscopy, and IHC expression for each marker was assessed semi-quantitatively on a 6-point scale: 0, none; 1, minimal; 2, mild; 3, moderate; 4, marked; 5, high.

Lung tissue RNA sequencing

Total RNA was extracted from frozen post-mortem lung samples (Banner Health Institute) (from *n* = 9 COVID-19 and *n* = 9 non-COVID pneumonia patients and *n* = 4 non-pneumonia participants) using RNeasy Plus mini kits (Qiagen, cat #74134) were used to extract RNA from 25 mg of frozen lung following a previously published protocol.[11] Tissues were lysed by mild sonication in an RNA-lysis buffer with 0.1M beta-mercaptoethanol and then processed according to the manufacturer's instructions. The concentration and purity of RNA samples were assessed using a Nanodrop spectrometer. RIN values were recorded from the Agilent's Bioanalyzer 2100 and RNA 6000 Nano kit (Agilent cat # 5067-1511). The library was constructed using the Illumina mRNA stranded kit (Novogene, Sacramento CA). Briefly, total RNA was reverse transcribed, and cDNA synthesised by LD-PCR amplification, purified (AMPure XP beads) and quantified (Qubit). cDNA samples were fragmented, end-repaired, polyA-tailed, ligated with adaptors, size selected, and polymerase chain-reaction (PCR)-

enriched. Sequencing was performed on an Illumina NovaSeq 6000 System to generate 30M 150-base-pair paired-end reads. RNA libraries were prepared in accordance with the NEBNext Ultra II Directional RNA Sample Preparation Protocol for Illumina Paired-End Multiplexed Sequencing. Sequenced libraries were assessed for quality using MultiQC based on STAR alignment against the GRCh38 ensembl (v100) human genome. Adapter trimming was performed using NGmerge, and Salmon was used for gene expression quantification using GRCh38 ensembl (v100) as a reference. The bioinformatics workflow was organised using Nextflow and Bioconda software management tools. Differential expression analyses were performed in R using the DESeq2 package with "apeglm" fold change shrinkage. The Benjamini–Hochberg method was used for multiple correction of *p* values.

Lung tissue single nucleus RNA sequencing

Single nucleus RNA sequencing datasets[12] from post-mortem lungs from patients with COVID-19 (n = 19, median age 72), and lung resection or biopsy tissue from non-COVID participants in the pre-COVID-19 era (n = 7, median age 70) were obtained from a published study.[4] Data was processed, and figures obtained using R language using Seurat (version 4.0.2)[13,14] and ggplot2 (version 3.3.5) R packages. Clinical details for COVID-19 patients and non-COVID participants are published.[4]

Endothelial cell RNA sequencing

Primary umbilical vein endothelial cells (HUVEC, Cambrex) maintained according to the manufacturer's instructions. HUVEC (passage 2, 2.5e5 cells/well, 6-well plates) were stimulated with 30 ng/mL purified recombinant IL-33^{red} [8] alone or in presence of vehicle control (PBS), 10 μg/mL anti-IL-33 mAb (tozorakimab,MEDI3506)[9] or 10 μg/mL human IgG1 isotype control mAb for 6 and 24 h. Total RNA was extracted with miRNeasy Mini Kit (Qiagen #217004) according to the manufacturer's instructions. RNA purity was determined using NanoDrop at 260 nm, and RNA quality assessed using the Agilent 2100 Bioanalyzer (Agilent Technologies). Libraries were prepared using KAPA Stranded mRNA-Seq Kit. mRNA was

enriched by ribosomal RNA depletion. Library quality was analysed using Agilent TapeStation analyzer and sequencing was performed with NovaSeq (2X100, 25 M reads per sample). FastQC files were generated and trimmed[15] and quality review was performed by FastQC [16] before and after quality trim. Reads were aligned with STAR aligner (version 2.6.1d, using the default settings) to the HG38 human genome and ENSEMBL94 annotation GTF file. After alignment and summarization with featureCounts of the Subread package (feature Counts release 1.6.3 and picard version 2.18.21), samples were normalised using voom (limma package) and differential gene expression analysis was carried out with limma *(*25605792*)* implemented in R. FDR adjusted p value < 0.05 was used as the cut-off (Benjamini-Hochberg method). Gene annotations were obtained by biomaRt.

Transcriptomic signature mapping

Signature mapping was performed using the Indication Discovery platform (AstraZeneca-DiseaseLinX, previously Onegevity Health LLC, New York, USA).[17,18] A library was generated of disease signatures from publicly available databases (764 signatures representing 310 diseases). Disease signatures were correlated with a HUVEC gene signature gene expression modified by 10 μg/mL tozorakimab with 30 ng/mL IL-33^{red} *versus* 10 mg/mL isotype antibody with 30 ng/mL IL-33^{red} at 6 and 24 hours (adj p value < 0.05) (Supplementary Table 10b). A modified Kolmogorov-Smirnov algorithm was used to generate adjusted connectivity scores (normScore; FDR<0.01). Negative connectivity scores (<0) indicate a negative correlation of the HUVEC tozorakimab signature with disease signatures.

Bioinformatic pathways and network analyses

Protein interaction network analysis was performed using Cytoscape (version 3.8.0)[19] and StringApp plugin[20] using a confidence score (cutoff 0.7, high confidence). Gene Ontology (GO) and KEGG enrichment analyses were performed using R and clusterProfiler[21] (adj p value < 0.05).

Integrated omics analyses

Integrated omics and network proximity associations were performed using the HUVEC transcriptomics signature (6 and 24 hours with IL-33^{red} + tozorakimab *versus* IL-33^{red} + isotype mAb, adj p value < 0.05, Supplementary Table 10b) and serum proteins from COVID-19 patients that associated with poor clinical outcomes (adj p value < 0.05, Supplementary Fig. 16 and Supplementary Table 7). Two network proximity-based methods (size of the largest connected component (LCC) and the average shortest path length (ASPL)) were used to quantify the proximity of the IL-33 transcriptomics signature and COVID-19 serum protein network compared with a reference distance distribution from randomly generated networks of similar size.

REFERENCES

1.Burke H, Freeman A, Dushianthan A, Celinski M, Batchelor J, Phan H *et al.* Research Evaluation Alongside Clinical Treatment in COVID-19 (REACT COVID-19): an observational and biobanking study. *BMJ Open* **11**, e043012 (2021).

2.R Core Team (2021). R: A language and environment for statistical computing. R Foundation for Statistical Computing, Vienna, Austria. https://www.R-project.org/. Accessed 02 February 2024.

3.Welch J, Dean J, Hartin J. Using NEWS2: an essential component of reliable clinical assessment. *Clin Med (Lond)* **22**, 509-513 (2022).

4.Melms JC, Biermann J, Huang H, Wang Y, Nair A, Tagore S *et al.* A molecular single-cell lung atlas of lethal COVID-19. *Nature* **595**, 114-119 (2021).

5.Persic L, Roberts A, Wilton J, Cattaneo A, Bradbury A, Hoogenboom HR. An integrated vector system for the eukaryotic expression of antibodies or their fragments after selection from phage display libraries. *Gene* **187**, 9-18 (1997).

6.Daramola O, Stevenson J, Dean G, Hatton D, Pettman G, Holmes W *et al.* A high-yielding CHO transient system: coexpression of genes encoding EBNA-1 and GS enhances transient protein expression. *Biotechnol Prog* **30**, 132-141 (2014).

7.Pace CN, Vajdos F, Fee L, Grimsley G, Gray T. How to measure and predict the molar absorption coefficient of a protein. *Protein Science* **4**, 2411-2423 (1995).

8.Cohen ES, Scott IC, Majithiya JB, Rapley L, Kemp BP, England E *et al.* Oxidation of the alarmin IL-33 regulates ST2-dependent inflammation. *Nat Commun* **6**, 8327 (2015).

9.England E, Rees DG, Scott IC, Carmen S, Chan DTY, Chaillan Huntington CE *et al.* Tozorakimab (MEDI3506): an anti-IL-33 antibody that inhibits IL-33 signalling via ST2 and RAGE/EGFR to reduce inflammation and epithelial dysfunction. *Sci Rep* **13**, 9825 (2023).

10.Strickson S, Houslay KF, Negri VA, Ohne Y, Ottosson T, Dodd RB *et al.* Oxidised IL-33 drives COPD epithelial pathogenesis via ST2-independent RAGE/EGFR signalling complex. *Eur Respir J* **62**, (2023).

11.Walker DG, Whetzel AM, Serrano G, Sue LI, Lue LF, Beach TG. Characterization of RNA isolated from eighteen different human tissues: results from a rapid human autopsy program. *Cell Tissue Bank* **17**, 361-375 (2016).

12.Single cell broad institute. https://singlecell.broadinstitute.org/single_cell/study/SCP1219 Accessed 02 February 2024

13. Hao Y, Hao S, Andersen-Nissen E, Mauck WM, 3rd, Zheng S, Butler A *et al.* Integrated analysis of multimodal single-cell data. *Cell* **184**, 3573-3587.e3529 (2021).

14. Stuart T, Butler A, Hoffman P, Hafemeister C, Papalexi E, Mauck WM, 3rd *et al.* Comprehensive Integration of Single-Cell Data. *Cell* **177**, 1888-1902.e1821 (2019).

15. Babraham bioinformatics. https://singlecell.broadinstitute.org/single_cell/study/SCP1219. Accessed 02 February 2024

16. Babraham bioinformatics. https://www.bioinformatics.babraham.ac.uk/projects/fastqc/. Accessed 02 February 2024

17. Dudley JT, Sirota M, Shenoy M, Pai RK, Roedder S, Chiang AP *et al.* Computational repositioning of the anticonvulsant topiramate for inflammatory bowel disease. *Sci Transl Med* **3**, 96ra76 (2011).

18. Subramanian A, Narayan R, Corsello SM, Peck DD, Natoli TE, Lu X *et al.* A Next Generation Connectivity Map: L1000 Platform and the First 1,000,000 Profiles. *Cell* **171**, 1437-1452.e1417 (2017).

19. Shannon P, Markiel A, Ozier O, Baliga NS, Wang JT, Ramage D *et al.* Cytoscape: a software environment for integrated models of biomolecular interaction networks. *Genome Res* **13**, 2498-2504 (2003).

20. Doncheva NT, Morris JH, Gorodkin J, Jensen LJ. Cytoscape StringApp: Network Analysis and Visualization of Proteomics Data. *J Proteome Res* **18**, 623-632 (2019).

21. Wu T, Hu E, Xu S, Chen M, Guo P, Dai Z *et al.* clusterProfiler 4.0: A universal enrichment tool for interpreting omics data. *Innovation (Camb)* **2**, 100141 (2021).

22. Schupp JC, Adams TS, Cosme C, Jr., Raredon MSB, Yuan Y, Omote N *et al.* Integrated Single-Cell Atlas of Endothelial Cells of the Human Lung. *Circulation* **144**, 286-302 (2021).

Supplementary Fig. 1. Commercial IL-33 assays have poor selectivity and sensitivity.

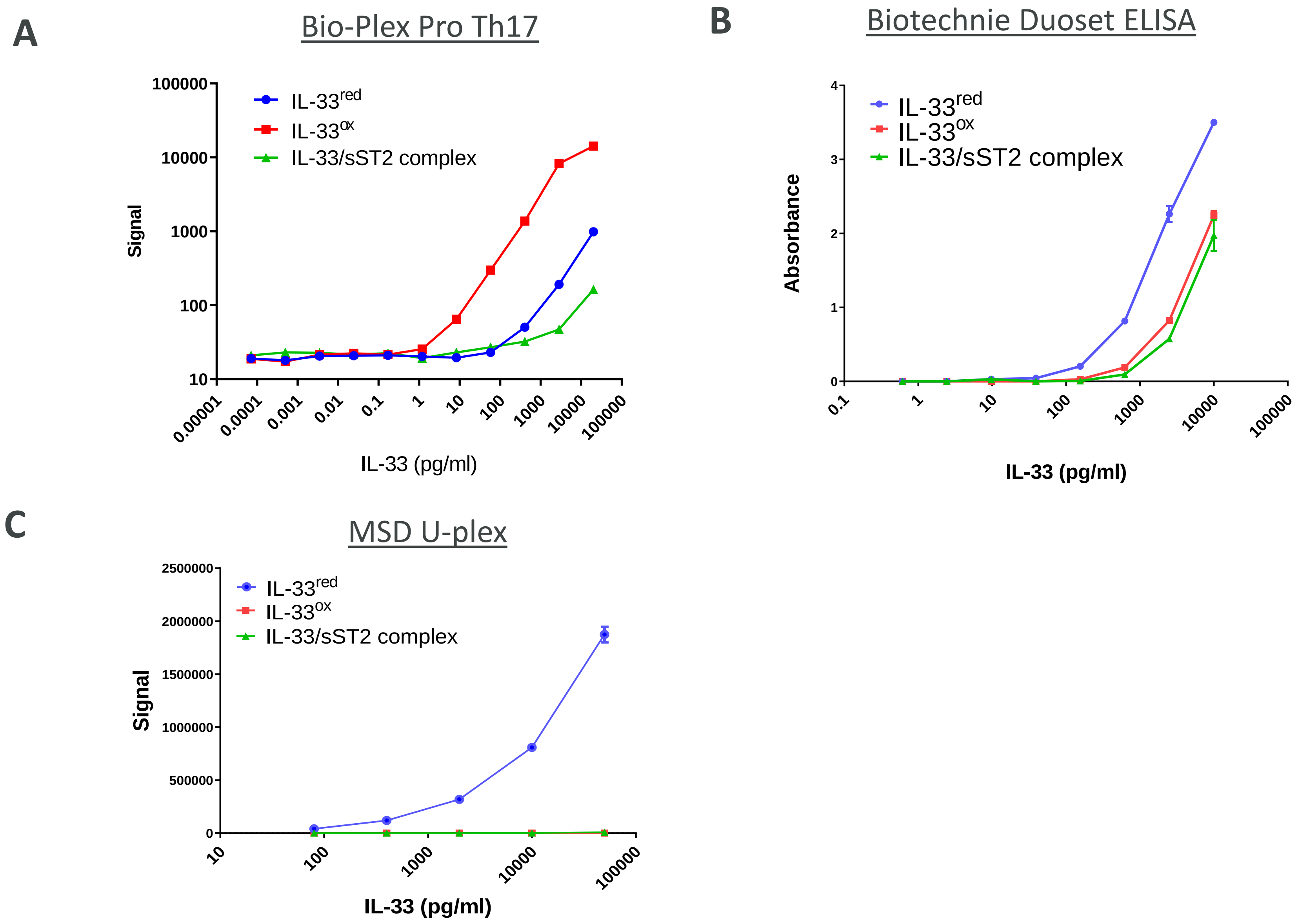

Measurement of recombinant human $IL\text{-}33^{red}$, $IL\text{-}33^{ox}$ and IL-33/sST2 complex standards in commercial (a) Bio-Plex Pro Th17, (b) Biotechnie R&D systems DuoSet ELISA and (c) Mesoscale Discovery (MSD) U-plex IL-33 immunoassays.

Supplementary Fig. 2. Summary of IL-33 hybridoma and anti-IL-33 mAb screening campaign.

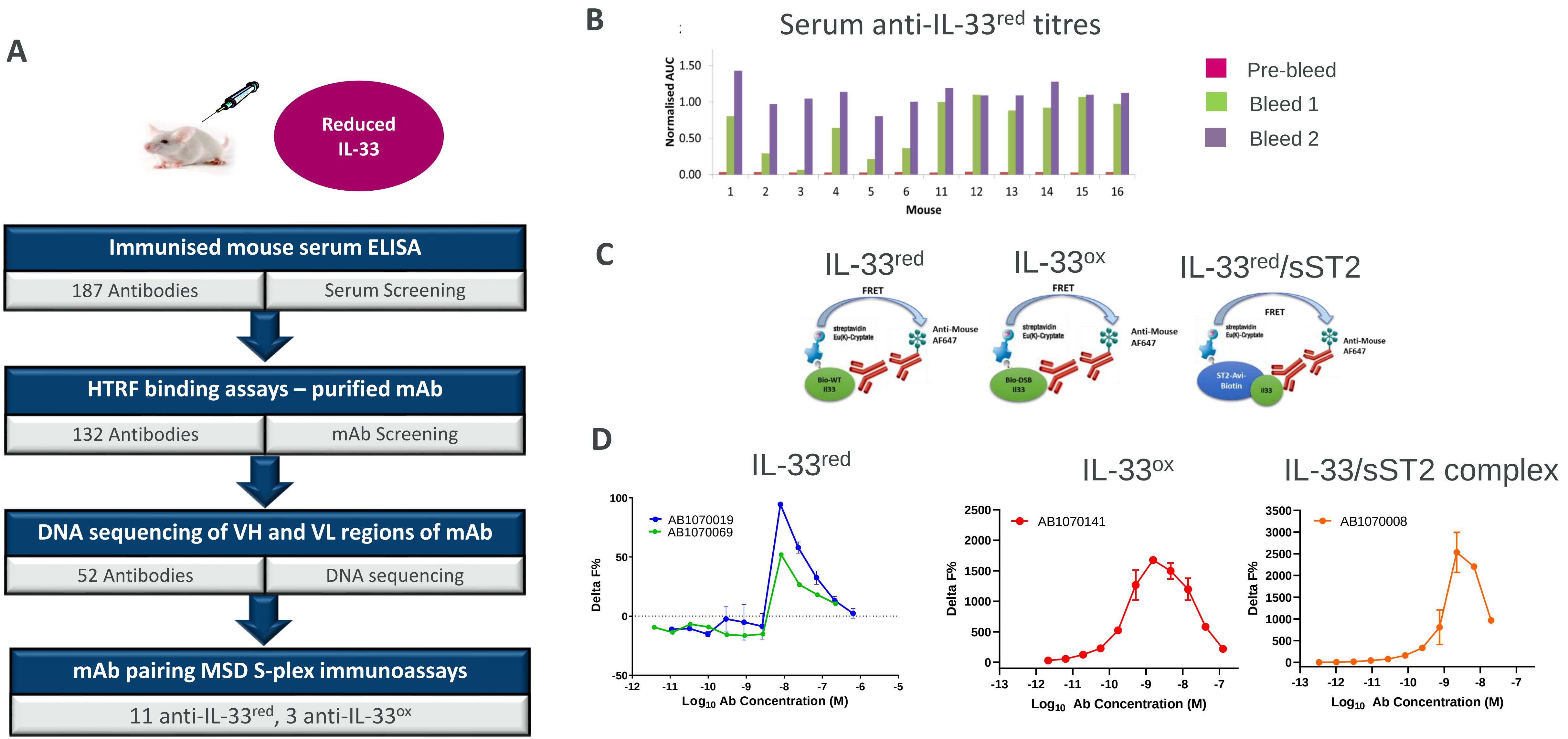

(a), summary of hybridoma screening campaign for identification of anti-IL-33^{red} and anti-IL-33^{ox} monoclonal antibodies (mAb). (b) example of serum antibody titres (anti-IL-33^{red} mAb) pre-bleed and at bleed 1 and 2; normalised area under the curve (AUC). (c) schematic diagram of homogeneous time resolved fluorescence energy transfer (HTRF) assays for IL-33^{red}, IL-33^{ox} and IL-33/ST2 complex. Fluorescence resonance energy transfer (FRET); Europium cryptate (Eu-K cryptate). Biotinylated IL-33^{red} (Bio-WT IL-33); biotinylated IL-33^{ox} (Bio-DSB IL-33); biotinylated-Avi-tagged sST2 (ST2-Avi-Bio). (d), Examples of IL-33^{red}, IL-33^{ox} and IL-33/sST2 complex HTRF assay data for selected anti-IL-33 mAb.

Supplementary Fig. 3. High sensitivity immunoassays for IL-33^{red}, IL-33^{ox} and IL-33/sST2 complexes.

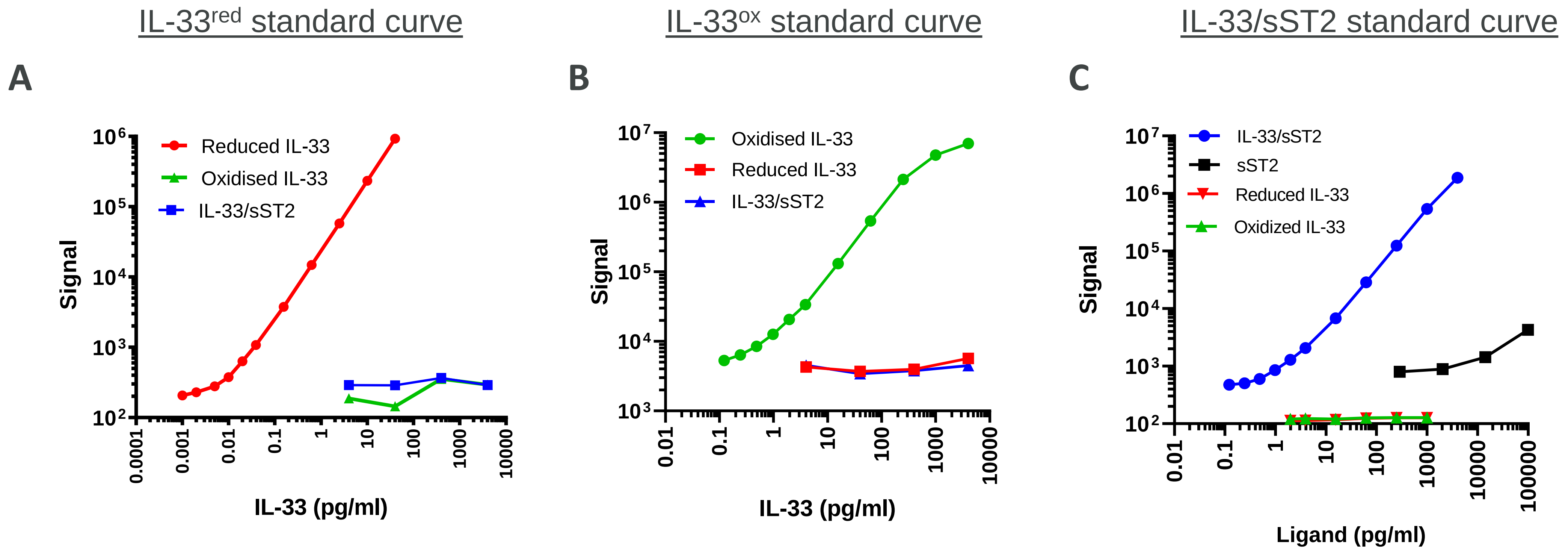

Measurement of recombinant human IL-33^{red}, IL-33^{ox}, IL-33/sST2 complex and sST2 standards in high sensitivity Mesoscale Discovery (MSD) S-plex IL-33 immunoassays

Supplementary Fig. 4. IL-33/sST2 complex is the circulating form of IL-33.

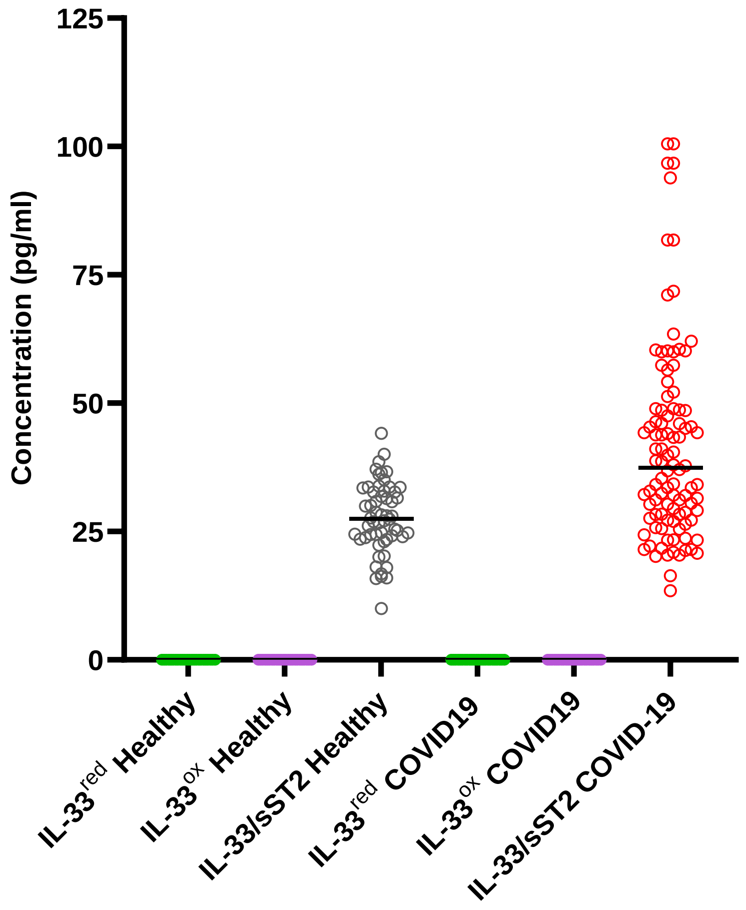

Measurement of IL-33^{red}, IL-33^{ox} and IL-33/sST2 complex in serum from healthy individuals (*n* = 56) and patients hospitalised with COVID-19 (Southampton cohort, *n* = 100 patients). A dot represents individual patient data; black lines represent median values.

Supplementary Fig. 5. Presage sST2 immunoassay detects sST2 in presence or absence of IL-33^{red}.

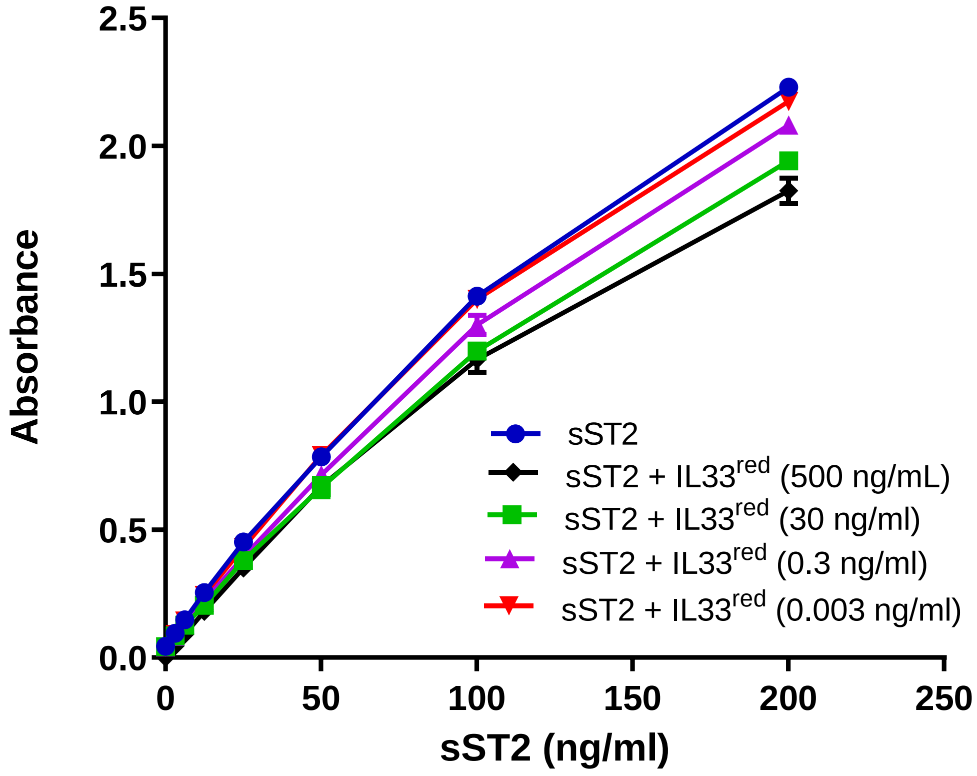

Measurement of recombinant sST2 standard in Presage immunoassay (Critical Diagnostics) in the presence and absence of recombinant IL-33^{red}.

Supplementary Fig. 6. Levels of IL-33/sST2 complex and sST2 are poorly correlated in serum from patients with COVID-19.

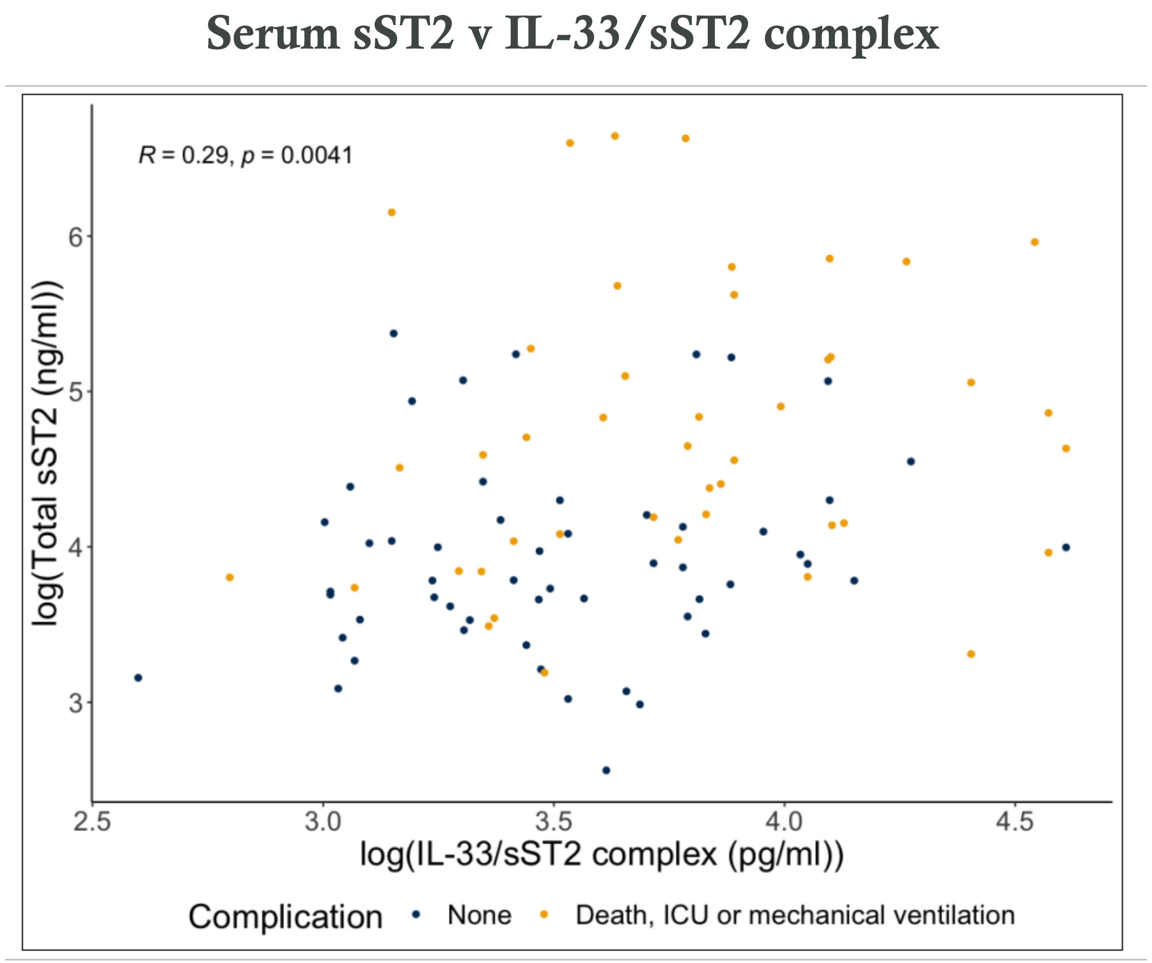

Pearson's correlation of levels of serum IL-33/sST2 complex and sST2 in patients hospitalised with COVID-19 (n = 100) with and without the composite clinical end point (death, need for intensive care (ICU) or mechanical ventilation) Pearson's correlation coefficient (R); p value (p).

Supplemental Fig. 7. sST2 levels are lower in nasal mucosal lining fluid compared to serum.

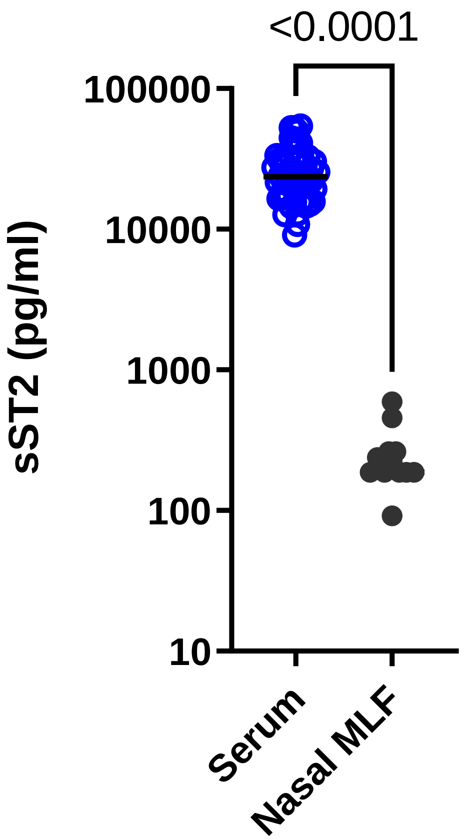

Measurement of sST2 in serum (n = 50) and nasal mucosal lining fluids (MLF) (n = 14) from healthy participants using Presage immunoassay.

Supplementary Fig. 8. IL-33^{red} and IL-33^{ox} in nasal MLF do not correlate with circulating IL-33/sST2 complex in patients with COVID-19.

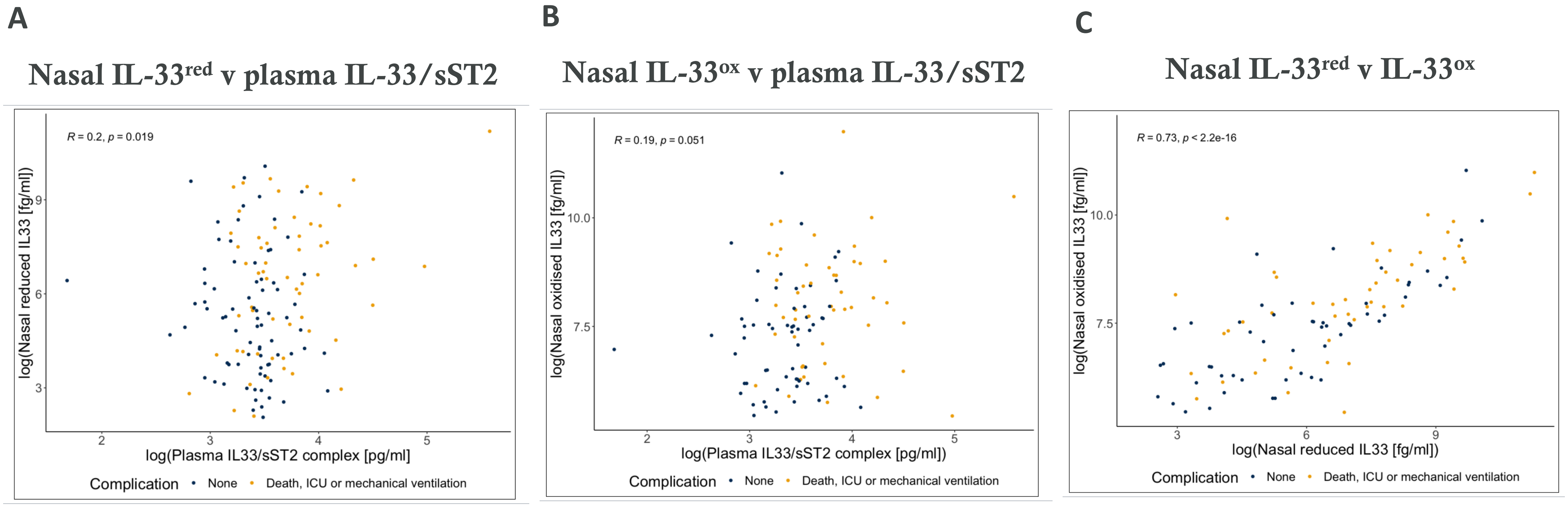

Pearson's correlation of levels of nasal IL-33^{red}, IL-33^{ox} and serum IL-33/sST2 complex from patients hospitalised with COVID-19 (*n* = 100) with and without composite clinical end point (death, need for intensive care (ICU) or mechanical ventilation) Pearson's correlation coefficient (R); *p* value (p).

Supplementary Fig. 9. Correlation of serum IL-33/sST2 complex and sST2 with clinical parameters in patients with COVID-19.

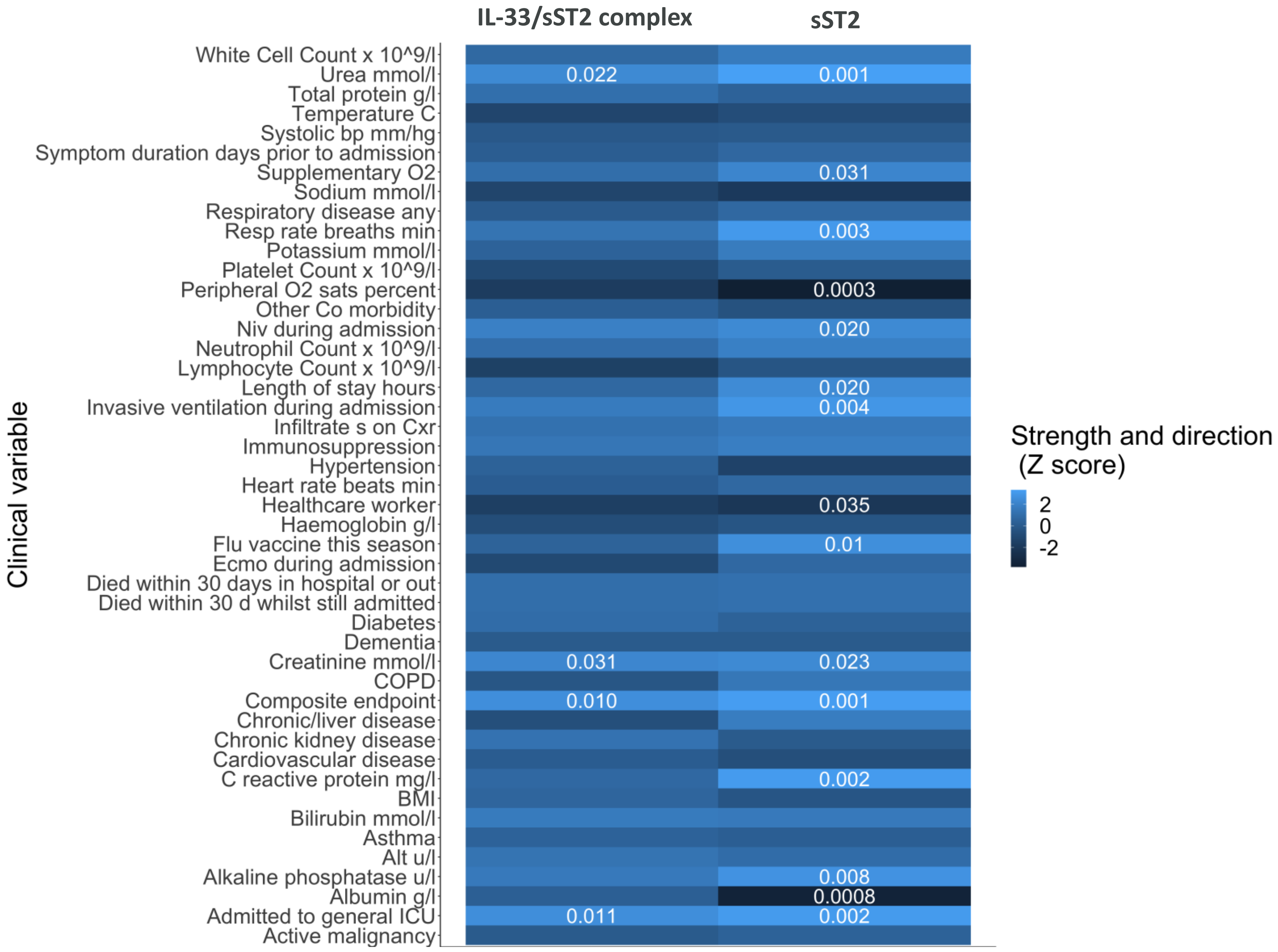

Heat map showing the associations of IL-33/sST2 complex and sST2 with clinical parameters in patients hospitalised with COVID-19. Aa linear regression (for continuous traits) or a logistic regression for binary traits were performed to derive p values and z scores (estimate/standard error). *P* values < 0.05 are shown; strength and direction of association are shown as a heat map (Z-score).

Supplementary Fig. 10. Correlations of IL-33/sST2 complex and sST2 levels with other serum mediators in patients with COVID-19.

Heat maps showing the correlation of IL-33/sST2 and sST2 with other serum mediators in patients hospitalised with COVID-19 derived from O-link proteomics analyses. Correlation coefficients (R) are shown; * indicates a p value < 0.05.

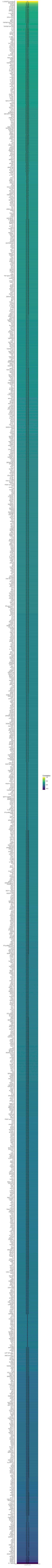

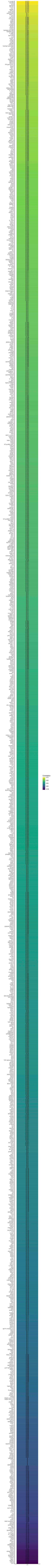
Correlation
0.75
0.50
0.25
0.00
-0.25

Supplementary Fig. 11. Correlations of semi-quantitative immunostaining for IL-33 and different cell markers in lung tissues.

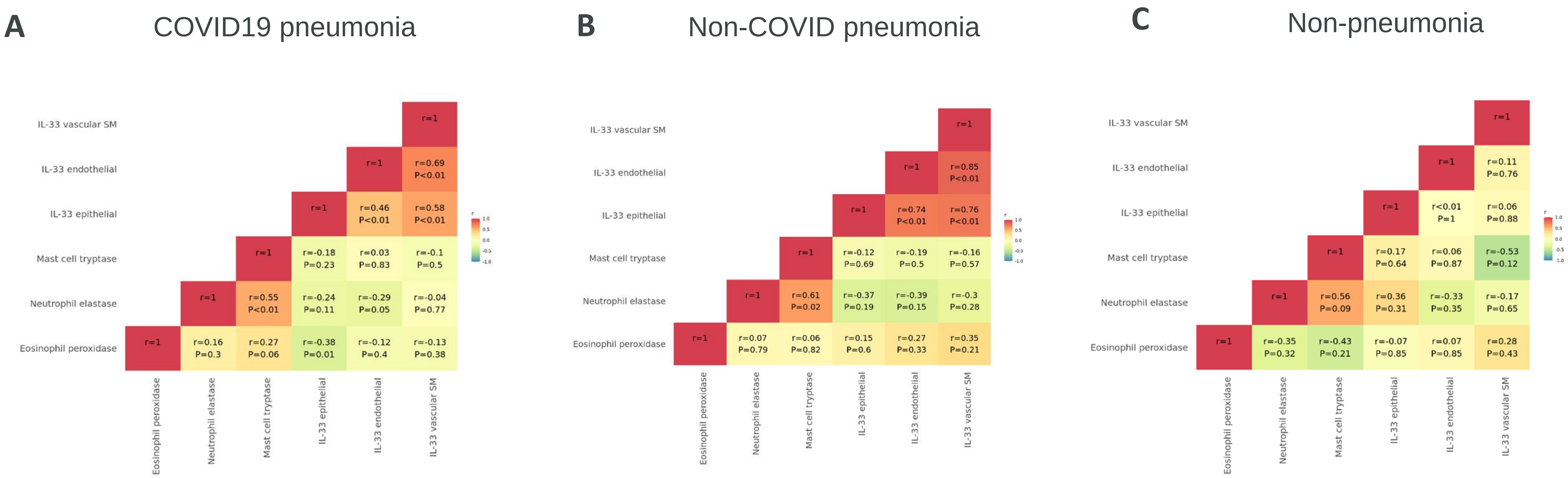

Correlations of semi-quantitative immunostaining scores for IL-33 and different cell markers in post-mortem lung tissue from COVID-19 pneumonia, non-COVID pneumonia and non-pneumonia patients. COVID-19 pneumonia (a), non-COVID pneumonia (b) and non-pneumonia (c). The numbers in the heatmap are correlation coefficients (r) with *p* values (P).

Supplementary Fig. 12. Gene expression levels for *IL1RL1*, *AGER* and *IL33* in lung tissues.

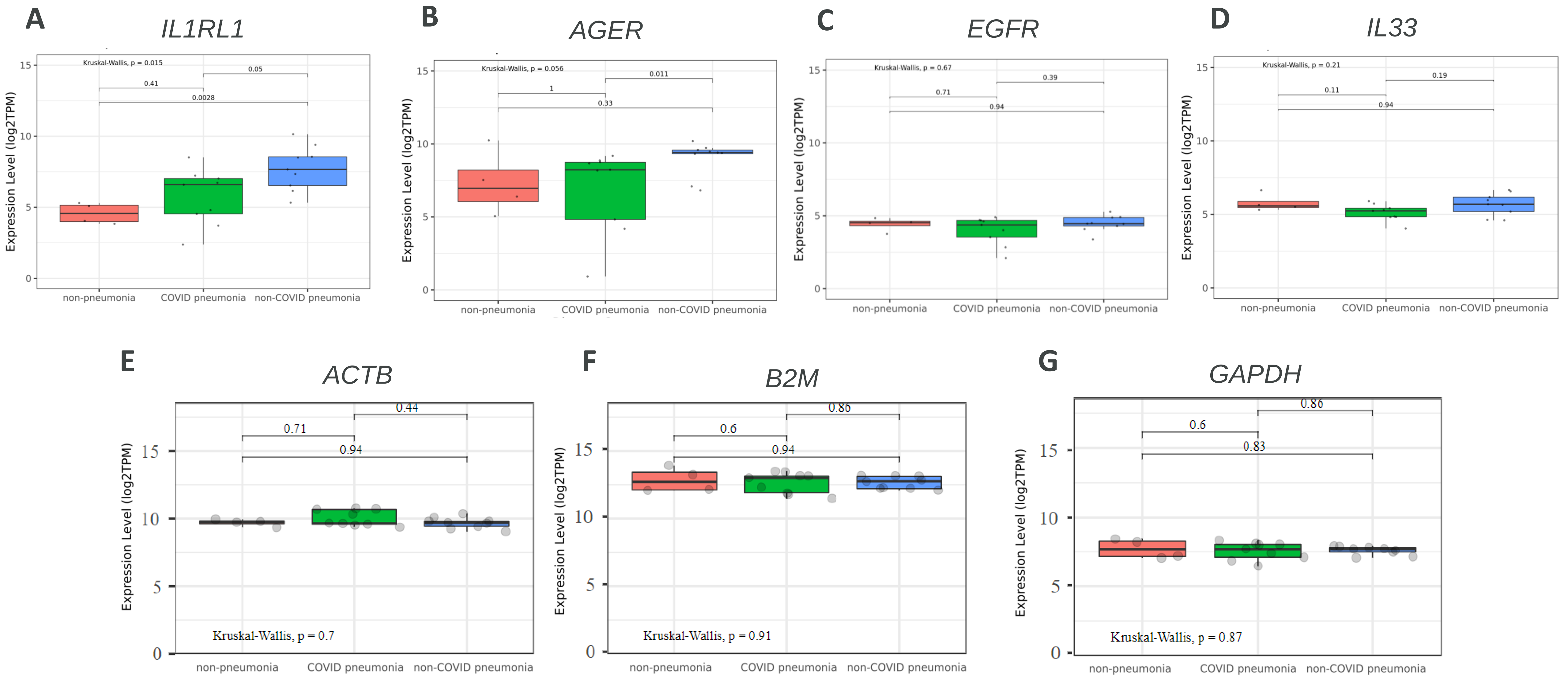

Quantification of gene expression levels for *IL1RL1* (a), *AGER* (b), *EGFR* (c), *IL33* (d), *ACTB* (e), *BSM* (f) and *GAPDH* (g) in post-mortem lung tissues from COVID-19 (*n* = 9) and non-COVID pneumonia (*n* = 9) patients and non-pneumonia participants (*n* = 4). Dots represent individual data points. Expression levels are shown as log2TPM= transcripts per million (normalised per individual); *p* values are shown above datasets Kruskal-Wallis test; error bars are standard deviations

Supplementary Fig. 13. Single nucleus expression profiles for *EGFR, AGER, IL33 and IL1RL1* in lung tissues.

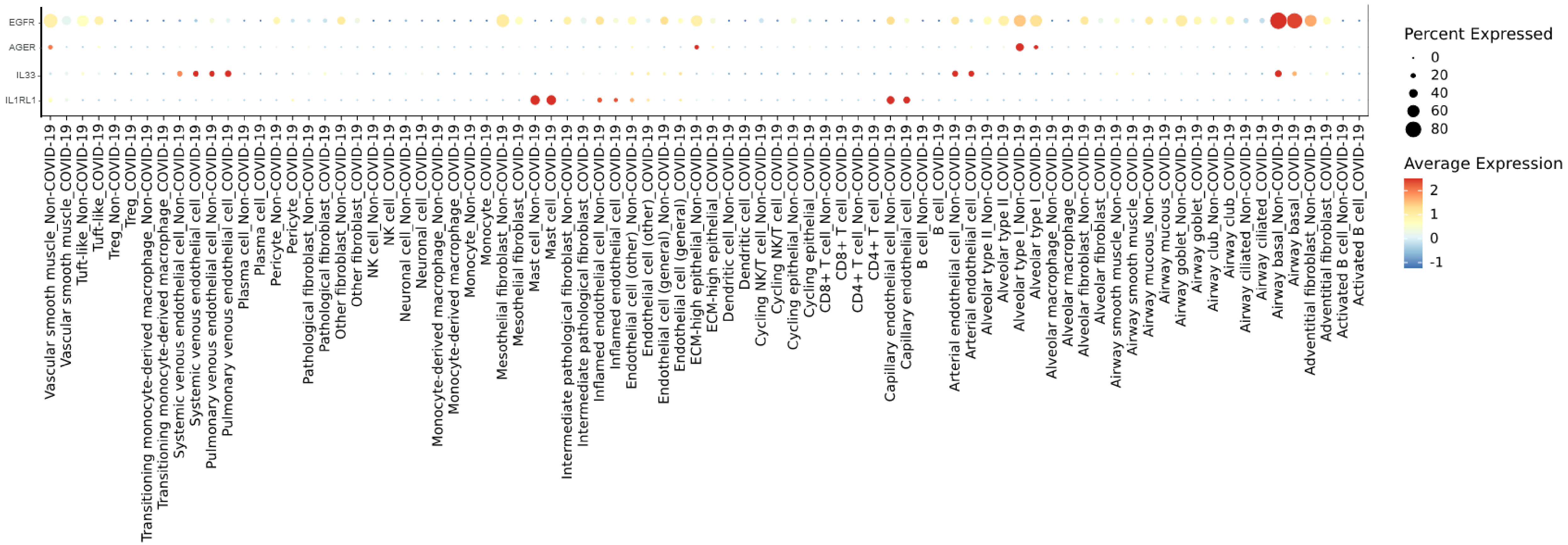

Dot plot showing the frequency and average scaled expression profiles of *EGFR*, *AGER*, *IL33* and *IL1RL1* in lung tissues from COVID-19 patients and non-COVID participants. Dot size represents the percentage of cells expressing each gene.

Supplementary Fig. 14. Comparison of the highest expressed gene markers in aerocytes with other endothelial cell types.

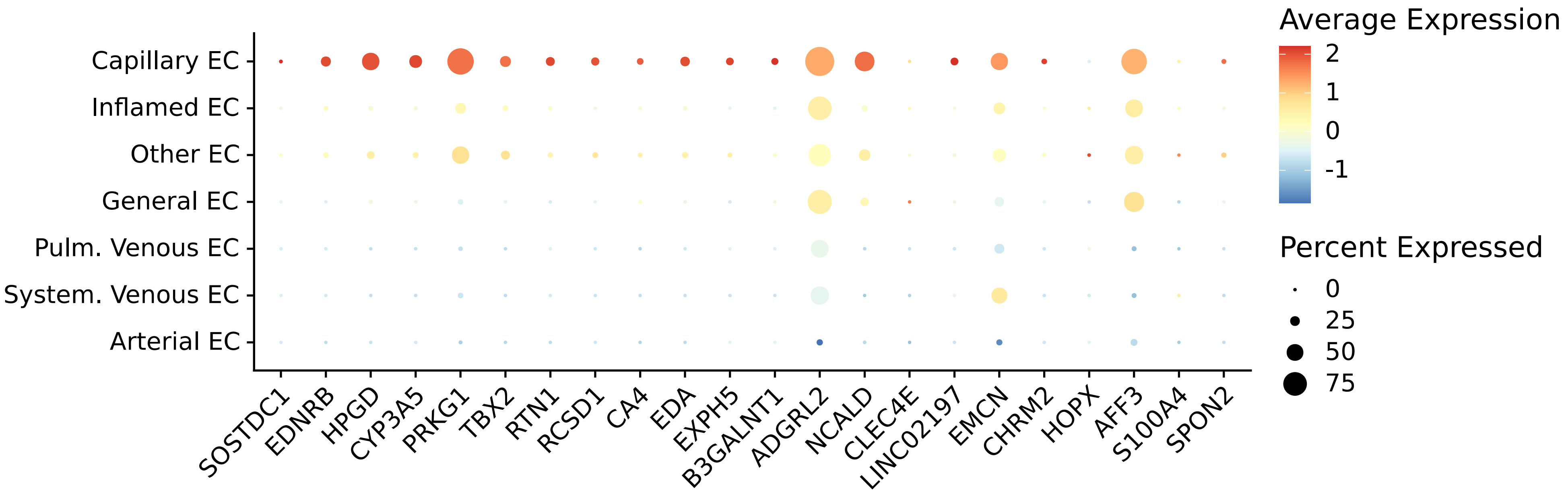

Dot plot showing the frequency and averaged scaled expression of the highest expressed gene markers for aerocytes compared to other endothelial cell types [4,24]. Dot size represents the percentage of cells expressing each gene. EC: endothelial cells.

Supplementary Fig. 15. Network map of differential gene expression in IL-33 stimulated endothelial cells treated with tozorakimab.

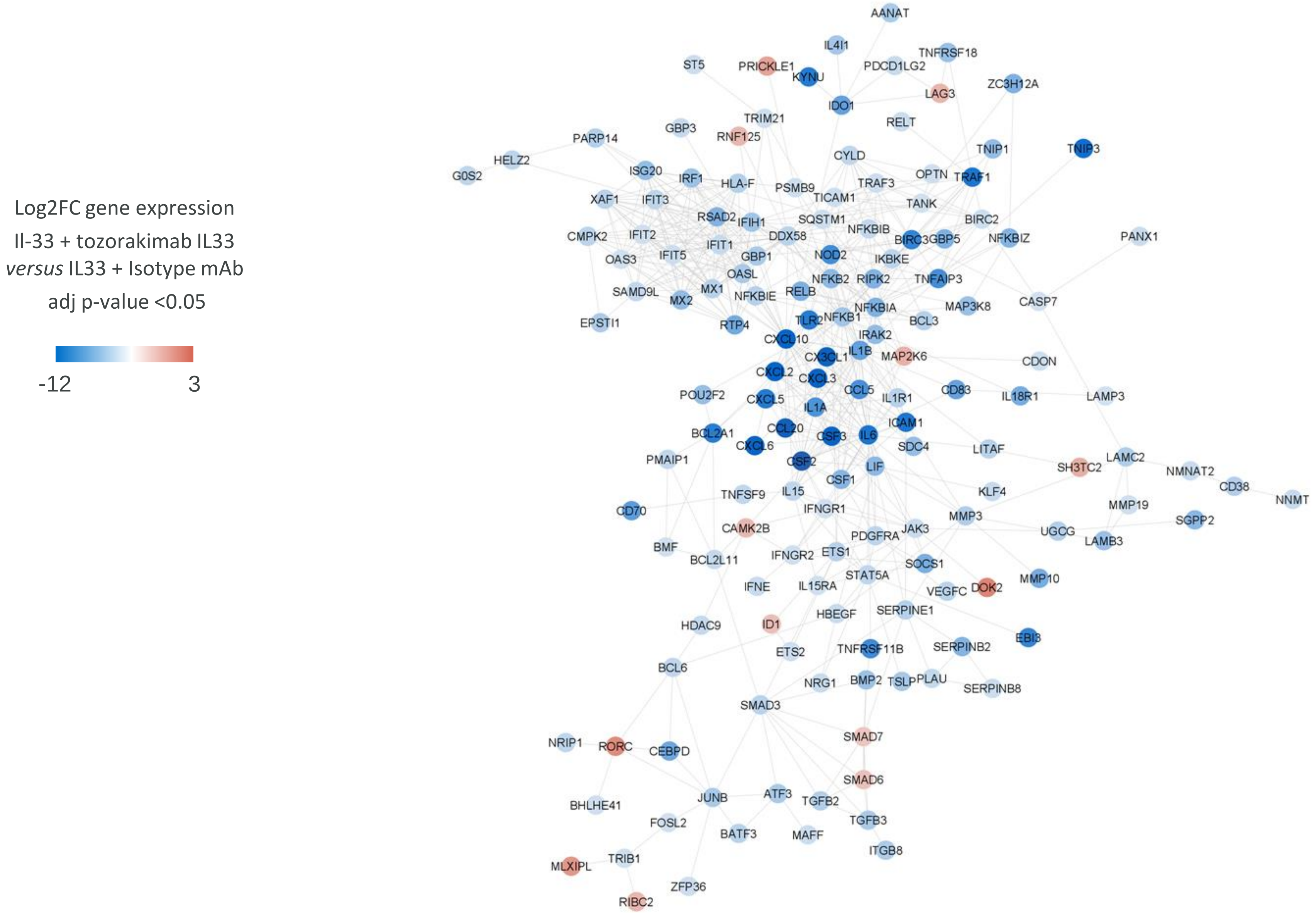

Network map of differential gene expression in HUVECs treated for 6 and 24 hours with IL-33^{red} + tozorakimab IL33 *versus* IL33 + isotype mAb. Log2FC gene expression changes with adjusted *p* value < 0.05 are shown.

Supplementary Fig. 16. Network map of serum proteins associated with poor clinical outcomes in patients with COVID-19.

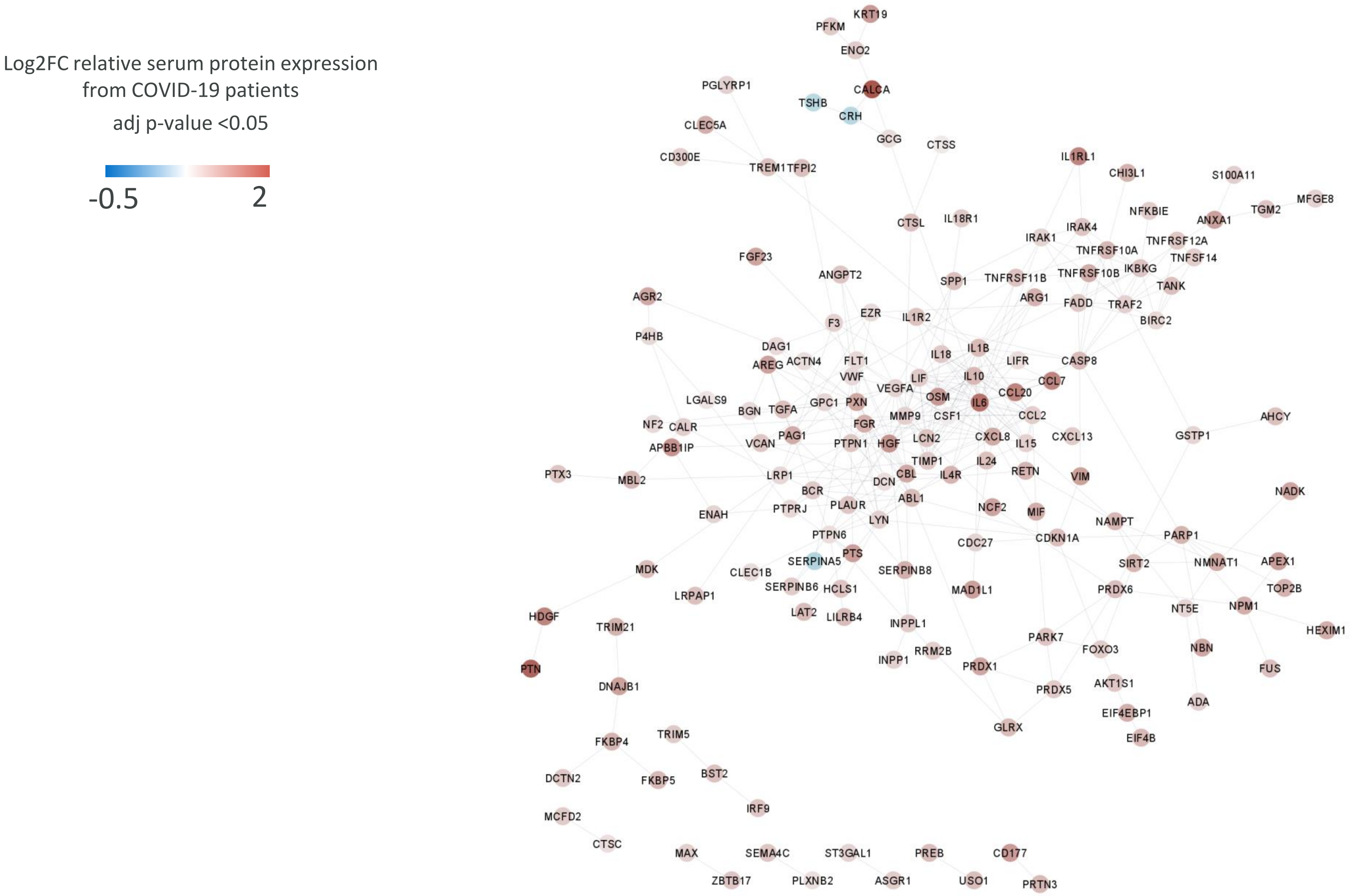

Network map of relative serum protein expression in COVID-19 patients associated with poor clinical outcomes (composite endpoint of death, need for intensive care or mechanical ventilation). Log2FC relative protein expression with adjusted *p* value < 0.05 are shown.

Supplementary Table 1. Demographics and clinical characteristics of the Southampton cohort of COVID-19 patients.

	Whole cohort (N = 100)	Presence composite endpoint (N = 44)	Univariate odds ratio (95CI) composite endpoint	Death (N = 17)	Univariate odds ratio (95CI) death
**Sex**					
Male	60.00% (n = 100)	61.36% (n = 44)	1.11 (0.49, 2.50)	58.82% (n = 17)	0.94 (0.33, 2.83)
Female	40.00% (n = 100)	38.64% (n = 44)	Ref	41.18% (n = 17)	Ref
**Age**					
Mean age	61.21 ± 17.18	62.82 ± 17.81	1.01 (0.99, 1.03)	81.35 ± 10.09	1.14 (1.08, 1.23)
Age ≥ 70 years old	36 (36.00%)	15 (34.09%)	0.86 (0.37, 1.96)	15 (88.24%)	22.1 (5.65, 148)
Age < 70 years old	64 (64.00%)	29 (65.91%)	Ref	2 (11.76%)	Ref
**Current Smoker**					
Yes	5 (5.00%)	2 (4.55%)	0.83 (0.11, 2.25)	1 (5.88%)	1.48 (0.07, 11.1)
No	83 (83.00%)	37 (84.09%)	Ref	12 (70.59%)	Ref
Unknown	12 (12.00%)	5 (11.36%)	0.89 (0.25, 3.01)	4 (23.53%)	2.96 (0.70, 11.1)
**Ethnicity**					
White	64 (64.00%)	29 (65.91%)	1.70 (0.75, 3.89)	15 (88.24%)	5.20 (1.35, 34.4)
Black and ethnic minorities	36 (36.00%)	15 (34.09%)	Ref	2 (11.76%)	Ref
**Past Medical History**					
Hypertension	41 (41.00%)	21 (47.73%)	1.70 (0.75, 3.89)	13 (76.47%)	5.92 (1.89, 22.6)
Cardiac disease	21 (21.00%)	10 (22.73%)	1.25 (0.47, 3.32)	8 (47.06%)	4.58 (1.48, 14.3)
COPD	9 (9.00%)	6 (13.64%)	2.89 (0.71, 14.4)	5 (29.41%)	7.92 (1.86, 36.2)
Asthma	17 (17.00%)	8 (18.18%)	1.20 (0.41, 3.46)	4 (23.53%)	1.59 (0.40, 5.35)
Chronic Respiratory Disease	27 (27.00%)	14 (31.82%)	1.62 (0.66, 3.99)	10 (58.82%)	5.29 (1.78, 16.6)
Chronic Kidney disease	6 (6.00%)	4 (9.09%)	2.79 (0.52, 20.9)	3 (17.65%)	5.50 (0.94, 32.5)
Liver Disease	3 (3.00%)	1 (2.27%)	0.65 (0.03, 6.97)	0 (0.00%)	-
Diabetes	23 (23.00%)	12 (27.27%)	1.60 (0.62, 4.15)	8 (47.06%)	3.85 (1.26, 11.8)
Malignancy	8 (8.00%)	4 (9.09%)	1.34 (0.30, 6.00)	4 (23.53%)	5.85 (1.25, 27.7)
Dementia	10 (10.00%)	4 (9.09%)	0.84 (0.20, 3.16)	4 (23.53%)	3.74 (0.86, 15.0)
**Presentation to Hospital**					
Median day of presentation (LQ, UQ)	7.00 (4.00, 10.00)	7.00 (2.00, 10.25)	1.02 (0.95, 1.09)	5.00 (0.00, 8.00)	0.98 (0.89, 1.07)
NEWS2 Score at admission to hospital Mean(±SD)	97; 5.13 ± 2.72	42; 6.10 ± 2.41	1.29 (1.10, 1.55)	16; 5.38 ± 2.36	1.04 (0.85, 1.28)
**Admission COVID19 Blood panel**					
Neutrophil Count 109L Median (IQR)	99; 5.60 (3.90, 8.30)	43; 7.70 (4.75, 9.95)	1.28 (1.12, 1.49)	16; 7.60 (5.70, 12.15)	1.15 (1.01, 1.31)
Lymphocyte Count 109L Median (IQR)	99; 1.00 (0.80, 1.20)	43; 1.00 (0.70, 1.25)	0.86 (0.42, 1.03)	16; 0.85 (0.67, 1.02)	0.78 (0.21, 1.10)
Neutrophil/Lymphocyte ratio Median (IQR)	99; 5.75 (3.62, 9.24)	43; 7.70 (5.18, 12.33)	1.15 (1.06, 1.27)	16; 9.70 (7.37, 13.22)	1.13 (1.02, 1.25)
C-Reactive Protein mg/L Median (IQR)	99; 107.00 (50.50, 163.00)	43; 153.00 (85.00, 254.00)	1.01 (1.01, 1.02)	16; 117.00 (19.00, 349.75)	1.00 (1.00, 1.01)
Ferritin ug/L Median (IQR)	80; 628.00 (289.00, 1,410.00)	35; 1,197.00 (558.00, 2,002.00)	-	10; 466.50 (269.00, 1,737.00)	-
Lactate dehydrogenase U/L Median (IQR)	69; 759.00 (543.00, 1,038.00)	30; 977.50 (760.50, 1,274.00)	-	7; 772.00 (760.50, 917.50)	-
D-dimer ng/ml	77; 388.00 (231.00, 575.00)	34; 459.50 (295.00, 877.00)	-	9; 574.00 (304.00, 1,094.00)	-
High sensitivity Tropon I ng/L Median (IQR)	77; 10.00 (5.00, 25.00)	34; 17.50 (8.00, 67.75)	-	10; 106.00 (49.25, 286.00)	-
**Treatments and Outcomes (within 30 days of hospitalisation**					
Intensive care admission	30 (30.00%)	30 (68.18%)	-	3 (17.65%)	0.44 (0.10, 1.50)
ECMO during admission	1 (1.00%)	1 (2.27%)	-	0 (0.00%)	-
NIV during admission	22 (22.00%)	21 (47.73%)	-	1 (5.88%)	-
Invasive Venthilation during admission	18 (18.00%)	18 (40.91%)	-	3 (17.65%)	-
Death	17 (17.00%)	17 (38.64%)	-		-
Composite Endpoint	44 (44.00%)		-	17 (100.00%)	-

n = 100 patients

Supplementary Table 2. Demographics and clinical characteristics of the ISARIC4C cohort of COVID-19 patients.

	Whole cohort (N = 182)	Presence composite endpoint (N = 46)	Univariate odds ratio (95CI) composite endpoint	Death (N = 27)	Univariate odds ratio (95CI) death
**Sex**					
Female	72 (39.56%)	13 (28.26%)	Ref	8 (29.63%)	Ref
Male	110 (60.44%)	33 (71.74%)	1.95 (0.96, 4.13)	19 (70.37%)	1.67 (0.71, 4.27)
Not specified	0	0	NA	0	NA
**Age**					
Mean age	60.1 ± 16.5	62.3 ± 10.5	1.01 (0.99, 1.03)	65.4 ± 11.8	1.02 (1.00, 1.05)
Above 70	78.6 ± 7.9 (26.92%)	75.2 ± 6 (23.91%)	0.82 (0.36, 1.74)	75.2 ± 6 (40.74%)	2.22 (0.92, 5.23)
Below 70	53.1 ± 13.2 (73.08%)	58.1 ± 7.9 (76.09%)	1.22 (0.58, 2.75)	58.2 ± 9.6 (59.26%)	0.45 (0.19, 1.09)
**Current smoker**					
Yes	13 (7.14%)	4 (8.70%)	1.38 (0.35, 4.55)	2 (7.41%)	0.87 (0.13, 3.53)
No	127 (69.78%)	31 (67.39%)	Ref	22 (81.48%)	Ref
Unknown	42 (23.08%)	11 (23.91%)	1.1 (0.48, 2.40)	3 (11.11%)	0.37 (0.08, 1.14)
**Ethnicity**					
White	124 (68.13%)	25 (54.35%)	0.44 (0.22, 0.89)	18 (66.67%)	0.92 (0.40, 2.29)
Other	58 (31.87%)	21 (45.65%)	Ref	9 (33.33%)	Ref
**Past medical history**					
Hypertension	31 (17.03%)	7 (15.22%)	1 (0.33, 2.83)	2 (7.41%)	0.54 (0.08, 2.53)
Cardiac disease	38 (20.88%)	13 (28.26%)	2.01 (0.90, 4.38)	11 (40.74%)	3.55 (1.43, 8.69)
COPD	2 (1.10%)	1 (2.17%)	3.28 (0.13, 85.7)	1 (3.70%)	8.62 (0.32, 233)
Asthma	28 (15.38%)	3 (6.52%)	0.34 (0.08, 1.05)	2 (7.41%)	0.41 (0.06, 1.52)
Chronic respiratory disease	20 (10.99%)	6 (13.04%)	1.46 (0.49, 3.93)	4 (14.81%)	1.58 (0.42, 4.83)
Chronic kidney disease	16 (8.79%)	5 (10.87%)	1.53 (0.46, 4.50)	3 (11.11%)	1.42 (0.31, 4.84)
Liver disease	3 (1.65%)	0	0 (NA)	0	0 (NA)
Diabetes	21 (11.54%)	6 (13.04%)	1.21 (0.41, 3.20)	2 (7.41%)	0.57 (0.09, 2.15)
Malignancy	9 (4.95%)	0	0 (NA)	0	0 (NA)
Dementia	1 (0.55%)	0	0 (NA)	0	0 (NA)
**Admission COVID19 Blood Panel**					
Neutrophil count 109L	93; 5.86 (4.37, 7.60)	29; 5.62 (4.38, 7.60)	0.98 (0.90, 1.03)	14; 5.30 (4.18, 8.95)	0.99 (0.87, 1.05)
Lymphocyte count 109L	93; 0.99 (0.69, 1.40)	29; 0.84 (0.63, 1.18)	0.37 (0.13, 0.86)	14; 0.88 (0.51, 1.12)	0.27 (0.05, 0.88)
Neutrophil / Lymphocyte ratio	93; 5.67 (3.95, 8.98)	29; 6.19 (5.02, 8.81)	1.05 (0.98, 1.13)	14; 6.39 (4.23, 10.24)	1.06 (0.99, 1.15)
C-Reactive protein mg/L	79; 117.00 (59.85, 185.20)	27; 133.00 (79.00, 234.00)	1.01 (1.00, 1.01)	13; 188.40 (56.00, 233.00)	1 (1.00, 1.01)
Ferritin ug/L	8; 1,052.50 (478.00, 1,510.50)	7; 1,183.00 (717.50, 1,563.00)	1.45	2; 1,425.50 (1,304.25, 1,546.75)	1 (1.00, 1.00)
Lactate dehydrogenase ug/L	16; 447.50 (355.75, 602.25)	9; 600.00 (455.00, 729.00)	1.01 (1.00, 1.03)	2; 532.00 (493.50, 570.50)	1 (0.99, 1.01)
D-dimer mg/L	79; 3.55 (2.26, 5.77)	26; 5.97 (3.44, 8.54)	1.44 (1.20, 1.80)	20; 5.65 (3.25, 7.80)	1.3 (1.09, 1.57)
**Treatments and outcomes (within 30 days of hospitalisation)**					
Intensive care admission	26 (14.29%)	26 (56.52%)	-	7 (25.93%)	3.36 (1.06, 10.6)
ECMO during admission	3 (1.65%)	3 (6.52%)	-	1 (3.70%)	3.11 (0.14, 34.6)
NIV during admission	21 (11.54%)	13 (28.26%)	-	5 (18.52%)	2.34 (0.66, 7.60)
Invasive ventilation during admission	14 (7.69%)	14 (30.43%)	-	4 (14.81%)	2.95 (0.71, 10.6)
Death	27 (14.84%)	27 (58.70%)	-		-
Composite endpoint	46 (25.27%)		-	27 (100.00%)	-

n = 182 patients

Supplementary Table 3. Hybridoma-derived and commercial antibodies for IL-33 biomarker assay development.

Antibody ID	Source	Isotype	Specificity
AB1070008	Hybridoma	Mouse mAb - IgG1	IL-33^{red}
AB1070012	Hybridoma	Mouse mAb - IgG1	IL-33^{red}
AB1070018	Hybridoma	Mouse mAb - IgG1	IL-33^{red}
AB1070019	Hybridoma	Mouse mAb - IgG2b	IL-33^{red}
AB1070069	Hybridoma	Mouse mAb - IgG1	IL-33^{red}
AB1070031	Hybridoma	Mouse mAb - IgG1	IL-33^{red}
AB1070005	Hybridoma	Mouse mAb - IgG1	IL-33^{red}
AB1070007	Hybridoma	Mouse mAb - IgG1	IL-33^{red}
AB1070110	Hybridoma	Mouse mAb - IgG1	IL-33^{red}
AB1070013	Hybridoma	Mouse mAb - IgG1	IL-33^{red}
AB1070140	Hybridoma	Mouse mAb - IgG1	IL-33^{ox}
AB1070141	Hybridoma	Mouse mAb - IgG1	IL-33^{ox}
AB1070146	Hybridoma	Mouse mAb - IgG1	IL-33^{ox}
H338L293	Phage Display	Human mAb- IgG1	IL-33^{red}
AF3625	Immunisation	Goat polyclonal	IL-33 all forms
ALX-804-840 (Nessy-1)	Enzo Life Sciences	Mouse mAb – IgG1	IL-33^{ox}
IL33305B	Adipogen	Mouse mAb – IgG2a	IL-33^{ox}
IL-33 UPLEX capture	MSD	Mouse mAb – IgG1	IL-33^{red}
IL-33 UPLEX detect	MSD	Mouse mAb – IgG1	IL-33^{red}
MAB523	R&D systems	Mouse mAb – IgG1	ST2
AF523	R&D systems	Goat polyclonal	ST2
MAB5232	R&D systems	Rabbit mAb - IgG	ST2
MAB5231	R&D systems	Mouse mAb – IgG1	ST2

Summary of hybridoma-derived and commercial antibodies used for immunoassay development for IL-33^{red}, IL-33^{ox} and IL-33/sST2 complex including antibody identification (ID), source, antibody isotypes and IL-33 form specificity.

Supplementary Table 4. Affinity measurements of anti-IL-33 capture antibodies.

IL-33 form	$IL\text{-}33^{red}$			$IL\text{-}33^{ox}$			IL-33/sST2 complex		
mAb	K_D (nM)	k_a ($M^{-1}\ s^{-1}$)	k_d (s^{-1})	K_D (nM)	k_a ($M^{-1}\ s^{-1}$)	k_d (s^{-1})	K_D (nM)	k_a ($M^{-1}\ s^{-1}$)	k_d (s^{-1})
AB1070069	0.32	9.8×10^5	3.1×10^{-4}	-	-	-	-	-	-
AB1070141	-	-	-	1.8	1.9×10^5	3.4×10^{-4}	-	-	-
AB1070008	0.90	5.7×10^5	5.1×10^{-4}	-	-	-	26.5	1.3×10^4	3.4×10^{-4}

Summary of BIAcore affinity (Kd), on rate (Ka) and off rate (Ks) measurements for anti-IL-33 capture antibodies (AB1070069, AB1070141 and AB1070008) for recombinant $IL\text{-}33^{red}$, $IL\text{-}33^{ox}$ and IL-33/sST2 complex.

Supplementary Table 5. Summary of assay performance using linear dilution and parallelism, spike recovery and selectivity of IL-33^{red}, IL-33^{ox} and IL-33/sST2 complex assays.

A - Linear dilution and Parallelism

	Fold Dilution	Human Nasal MLF	
		Average % Recovery	% Recovery Range
Reduced IL-33	1	65	48 - 76
	2	83	63 - 96
	4	87	69 - 100
	8	100	100 - 100
	16	105	98 - 114
	32	107	97 - 116

	Fold Dilution	Human Nasal MLF	
		Average % Recovery	% Recovery Range
Oxidised IL-33	1	56	40 - 72
	2	76	65 - 86
	4	93	87 - 106
	8	100	100 - 100
	16	114	112 - 116
	32	119	113 - 125

	Fold Dilution	Human EDTA Plasma		Human Heparin Plasma		Human Serum	
		Average % Recovery	% Recovery Range	Average % Recovery	% Recovery Range	Average % Recovery	% Recovery Range
IL-33/sST2 complex	1	75	67 - 81	69	62 - 73	70	58 - 80
	2	89	83 - 92	85	82 - 88	88	81 - 96
	4	100	100 - 100	100	100 - 100	100	100 - 100
	8	110	102 - 114	113	108 - 119	109	107 - 114
	16	117	110 - 123	123	117 - 128	121	117 - 127

B - Spike Recovery

	Spike Level	Human Nasal MLF	
		Average % Recovery	% Recovery Range
Reduced IL-33	Spike	83	71 - 89

	Spike Level	Human Nasal MLF	
		Average % Recovery	% Recovery Range
Oxidised IL-33	Spike	80	76 - 83

	Spike Level	Human EDTA Plasma		Human Heparin Plasma		Human Serum	
		Average % Recovery	% Recovery Range	Average % Recovery	% Recovery Range	Average % Recovery	% Recovery Range
IL-33/sST2 complex	High Spike	79	72 - 90	79	75 - 89	76	71 - 84
	Mid Spike	78	74 - 85	75	69 - 80	76	73 - 81
	Low Spike	81	76 - 92	76	64 - 86	78	74 - 84

C - Selectivity

Reduced IL-33		
Sample	Tested Conc. (pg/mL)	% Non-Specificity
Reduced IL-33	5	100.00
Oxidised IL-33	100	0.02
IL-33/sST2	100	0.19
IL-18	100	0.02
IL-1a	100	0.02
IL-1b	100	0.02
IL-1RA	100	0.02
IL-25	100	0.02
IL-36γ	100	0.02
IL-5	100	0.02
IL-6	100	0.02
IL-8	100	0.02
RAGE	100	0.02
sST2	100	0.02
TSLP	100	0.02

Oxidised IL-33		
Sample	Tested Conc. (pg/mL)	% Non-Specificity
Oxidised IL-33	100	100.00
Reduced IL-33	100	0.07
IL-33/sST2	100	0.07
IL-18	100	0.07
IL-1a	100	0.07
IL-1b	100	0.07
IL-1RA	100	0.07
IL-25	100	0.07
IL-36γ	100	0.07
IL-5	100	0.07
IL-6	100	0.07
IL-8	100	0.07
RAGE	100	0.07
sST2	100	0.07
TSLP	100	0.07

IL-33/sST2 complex		
Sample	Tested Conc. (pg/mL)	% Non-Specificity
IL-33/sST2	100	100.00
Reduced IL-33	100	1.71
Oxidised IL-33	100	1.72
IL-18	100	1.67
IL-1a	100	1.60
IL-1b	100	1.53
IL-1RA	100	1.66
IL-25	100	1.63
IL-36γ	100	1.56
IL-5	100	1.61
IL-6	100	1.65
IL-8	100	1.72
RAGE	100	1.67
sST2	100	1.61
TSLP	100	1.61

IL-33^{red}, IL-33^{ox} and IL-33/sST2 complex assay performance using linear dilution and parallelism, spike recovery in serum, EDTA- and heparin-plasma and nasal mucosal lining fluids from *n* = 5 individuals, and analyte selectivity. (A) % average recovery and % recovery range at various fold dilutions of each sample matrix type. (B) % average recovery and % recovery range following spiking of recombinant human protein into each sample matrix type. (C) Tested concentrations and % non-selectivity was tested against a panel of recombinant human proteins. % recovery = (measured concentration/expected concentration) x100; % non-selectivity = (non-specific signal/specific signal) x 100.

Supplementary Table 6. Summary of IL-33 biomarker assays.

Assay Performance Summary

Assays	Capture Ab	Detection Ab	LLOD	ULOD	LLOQ	ULOQ	MRD Serum Plasma	MRD Nasal MLF	Accuracy Intra-run Recovery	Accuracy Inter-run Recovery	Precision Intra-run CV	Precision Inter-run CV
**IL-33^{red}**	AB1070069	AB1070019	0.001 pg/ml	20 pg/ml	0.005 pg/ml	16 pg/ml	NA	8	80-120%	80-120%	<20%	<20%
**IL-33^{ox}**	AB1070141	AF3625 R&D systems	0.02 pg/ml	100 pg/ml	0.1 pg/ml	100 pg/ml	NA	8	80-120%	80-120%	<20%	<20%
**IL-33^{red}/sST2**	AB1070008	BAF5232 R&D systems	0.2 U/ml	1000 U/ml	1 U/ml	1000 U/ml	4	NA	80-120%	80-120%	<20%	<20%

Summary of capture antibodies, detection antibodies and lower and upper limits of detection (pg/mL) for the IL-33^{red}, IL-33^{ox} and IL-33/sST2 complex MSD S-plex immunoassays.

Supplementary Table 7. Associations of serum proteins with poor patient outcomes in patients with COVID-19.

Serum COVID19
O-link dataset

Summary table of associations of serum proteins ($n = 1094$ proteins from O-link proteomics panels) with poor patient outcomes in hospitalised patients with COVID-19 ($n = 100$ patients, Southampton cohort) including protein assay, odds ratios (OR), p values, adjusted p values of serum proteins; Summary table is ordered by lowest to highest adjusted p values (significance adj p value < 0.05).

Supplementary Table 8. Patient demographics and pathology reports associated with lung tissue samples.

Banner health demographics

Summary table of post-mortem lung tissue histology and RNA samples (Banner Sun Health), and associated patient demographics (race, gender, age), SARS-CoV-2 tests, and pathology reports for COVID-19 (*n* = 15) and non-COVID pneumonia (*n* = 10) patients and non-pneumonia participants (*n* = 5).

Supplementary Table 9. Semi-quantitative scoring of immunohistochemical staining of lung tissue samples.

Histo #/Block ID	SID	Lobe	Disease Category	Eosinophil peroxidase (EPX)	Neutrophil elastase	Mast cell tryptase	IL-33 staining in bronchial and bronchiolar basal epithelial cells	IL-33 staining in endothelial cells	IL-33 staining in vascular smooth muscle cells
GHP002415-1A	1130	LL	COVID neg pneumonia	1.5	5.5	4.5	3.25	4.25	0
GHP002415-1B	1130	UL	COVID neg pneumonia	1.5	4.5	4.5	4.25	5.5	3.75
GHP002416-1A	1138	LL	COVID neg pneumonia	2.5	4.5	4.5	1.75	4.25	1.5
GHP002416-1B	1138	UL	COVID neg pneumonia	1.5	4.5	4.5	4.25	4.25	2.25
GHP002417-1A	1149	LL	COVID neg pneumonia	1.5	5.5	3.5	1.75	4.25	1.75
GHP002417-1B	1149	LL	COVID neg pneumonia	1.5	3.5	3.5	3.75	5.5	2.75
GHP002418-1B	1159	not specified	COVID neg pneumonia	2.5	3.5	3.5	N/A	4.25	2.25
GHP002418-1A	1159	LL	COVID neg pneumonia	2.5	4.5	3.5	5.5	5.5	4.25
GHP002419-1A	1165	not specified	COVID neg pneumonia	3.5	5.5	4.5	5.5	5.5	4.25
GHP002419-1B	1165	LL	COVID neg pneumonia	1.5	4.5	4.5	5.5	5.5	4.25
GHP002791-1A	1910	LLL	COVID neg pneumonia	3.5	4.5	4.5	3.75	5.5	3.25
GHP002792-1A	1905	LLL	COVID neg pneumonia	1.5	2.5	3.5	5.5	5.5	3.25
GHP002793-1A	1970	LLL	COVID neg pneumonia	2.5	3.5	3.5	4.75	5.5	3.25
GHP002794-1A	1968	LLL	COVID neg pneumonia	2.5	3.5	3.5	4.75	5.5	3.75
GHP002795-1A	1961	LLL	COVID neg pneumonia	2.5	3.5	3.5	4.25	5.5	3.75
GHP002425-1A	2035	LLL1	COVID pos pneumonia	2.5	5.5	3.5	4.25	3.25	2.75
GHP002425-1B	2035	RUL2	COVID pos pneumonia	1.5	2.5	4.5	5.5	5.5	1.75
GHP002426-1A	2044	LL	COVID pos pneumonia	0	4.5	3.5	5.5	5.5	3.75
GHP002426-1B	2044	RLL	COVID pos pneumonia	0	4.5	4.5	5.5	5.5	4.25
GHP002427-1A	2045	LLL1	COVID pos pneumonia	3.5	5.5	4.5	3.75	4.25	1.75
GHP002427-1B	2045	RUL2	COVID pos pneumonia	3.5	4.5	4.5	4.25	4.25	1.75
GHP002428-1A	2049	LLL1	COVID pos pneumonia	3.5	4.5	4.5	4.75	5.5	2.75
GHP002428-1B	2049	RUL2	COVID pos pneumonia	2.5	4.5	4.5	4.25	5.5	3.75
GHP002450-1A	2040	LLL	COVID pos pneumonia	3.5	4.5	4.5	4.25	4.25	1.75
GHP002450-1B	2040	LUL	COVID pos pneumonia	3.5	3.5	4.5	4.25	4.75	1.5
GHP002451-1A	2043	LLL	COVID pos pneumonia	0	4.5	4.5	3.25	4.25	0
GHP002451-1B	2043	LUL	COVID pos pneumonia	1.5	4.5	4.5	3.25	4.25	1.75
GHP002749-1A	2067	LUL 1	COVID pos pneumonia	3.5	2.5	3.5	4.25	4.75	3.25
GHP002749-1B	2067	LUL 2	COVID pos pneumonia	3.5	4.5	4.5	4.25	4.75	1.75
GHP002749-1C	2067	LUL 3	COVID pos pneumonia	3.5	3.5	4.5	4.25	4.75	2.75
GHP002749-1D	2067	LUL 4	COVID pos pneumonia	3.5	3.5	4.5	4.25	4.75	1.5
GHP002749-2A	2067	LLL1	COVID pos pneumonia	3.5	4.5	3.5	4.25	4.75	2.75
GHP002749-2B	2067	LLL2	COVID pos pneumonia	3.5	3.5	4.5	4.25	4.75	2.75
GHP002749-2C	2067	LLL3	COVID pos pneumonia	3.5	3.5	3.5	4.25	4.75	1.5
GHP002749-2D	2067	LLL4	COVID pos pneumonia	3.5	3.5	3.5	4.25	4.75	1.5
GHP002750-1A	2072	LUL1	COVID pos pneumonia	1.5	3.5	ND	4.25	4.25	1.5
GHP002750-1B	2072	LUL2	COVID pos pneumonia	3.5	3.5	2.5	4.25	4.25	1.75
GHP002750-1C	2072	LUL3	COVID pos pneumonia	2.5	3.5	3.5	4.25	4.25	1.5
GHP002750-1D	2072	LUL4	COVID pos pneumonia	2.5	3.5	2.5	4.25	4.25	1.75
GHP002750-2A	2072	LLL1	COVID pos pneumonia	1.5	3.5	2.5	4.25	4.25	1.75
GHP002750-2B	2072	LLL2	COVID pos pneumonia	1.5	3.5	2.5	4.25	4.25	1.75
GHP002750-2C	2072	LLL3	COVID pos pneumonia	0	1.5	1.5	N/A	4.75	1.5
GHP002750-2D	2072	LLL4	COVID pos pneumonia	1.5	3.5	2.5	4.75	4.25	2.75
GHP002751-1A	2073	LUL1	COVID pos pneumonia	2.5	ND	2.5	4.25	4.75	3.25
GHP002751-1B	2073	LUL2	COVID pos pneumonia	2.5	3.5	3.5	5.5	5.5	4.75
GHP002751-1C	2073	LUL3	COVID pos pneumonia	2.5	3.5	3.5	4.25	5.5	3.25
GHP002751-1D	2073	LUL4	COVID pos pneumonia	2.5	3.5	3.5	4.25	5.5	2.75
GHP002751-2A	2073	LLL1	COVID pos pneumonia	2.5	ND	3.5	4.25	5.5	3.25
GHP002751-2B	2073	LLL2	COVID pos pneumonia	3.5	4.5	ND	4.25	5.5	3.25
GHP002751-2C	2073	LLL3	COVID pos pneumonia	2.5	ND	ND	4.25	4.75	2.75
GHP002751-2D	2073	LLL4	COVID pos pneumonia	2.5	4.5	4.5	4.25	5.5	2.25
GHP002752-1A	2075	LUL1	COVID pos pneumonia	0	2.5	2.5	5.5	5.5	4.75
GHP002752-2A	2075	LLL1	COVID pos pneumonia	3.5	2.5	2.5	4.25	5.5	1.5
GHP002796-1A	2101	LLL	COVID pos pneumonia	3.5	3.5	4.5	4.25	5.5	3.75
GHP002797-1A	2105	LLL	COVID pos pneumonia	3.5	4.5	3.5	3.75	4.25	3.25
GHP002797-1B	2105	UL	COVID pos pneumonia	3.5	4.5	3.5	4.25	5.5	3.75
GHP002798-1A	2108	LL	COVID pos pneumonia	1.5	3.5	3.5	4.25	5.5	2.25
GHP002798-1B	2108	UL	COVID pos pneumonia	1.5	3.5	3.5	4.25	5.5	2.75
GHP002799-1A	2110	LL	COVID pos pneumonia	2.5	4.5	4.5	4.25	3.75	0
GHP002799-1B	2110	UL	COVID pos pneumonia	2.5	4.5	3.5	4.25	2.25	0
GHP002800-1A	2113	LL	COVID pos pneumonia	1.5	4.5	3.5	5.5	5.5	4.25
GHP002800-1B	2113	UL	COVID pos pneumonia	1.5	4.5	3.5	5.5	5.5	3.75
GHP002801-1A	2060	RLL	COVID pos pneumonia	2.5	5.5	4.5	3.75	3.25	0
GHP002801-1B	2060	RUL	COVID pos pneumonia	1.5	4.5	4.5	4.25	5.5	3.75
GHP002420-1A	1861	not specified	COVID neg non-pneumonia	1.5	2.5	3.5	5.5	5.5	2.75
GHP002420-1B	1861	LL	COVID neg non-pneumonia	2.5	2.5	2.5	5.5	5.5	4.25
GHP002421-1A	1944	not specified	COVID neg non-pneumonia	1.5	2.5	3.5	3.75	5.5	2.75
GHP002421-1B	1944	LL	COVID neg non-pneumonia	1.5	3.5	3.5	N/A	4.25	3.25
GHP002422-1A	1956	not specified	COVID neg non-pneumonia	2.5	2.5	3.5	4.25	5.5	4.25
GHP002422-1B	1956	RLL	COVID neg non-pneumonia	1.5	2.5	3.5	4.25	5.5	3.75
GHP002423-1A	1965	LL	COVID neg non-pneumonia	1.5	3.5	3.5	4.25	5.5	3.25
GHP002423-1B	1965	UL	COVID neg non-pneumonia	2.5	3.5	4.5	5.5	5.5	2.75
GHP002424-1A	1976	not specified	COVID neg non-pneumonia	0	3.5	4.5	5.5	5.5	3.25
GHP002424-1B	1976	LL	COVID neg non-pneumonia	1.5	3.5	3.5	5.5	5.5	4.25

Heat map of semi-quantitative scoring of immunohistochemical staining of post-mortem lung tissues (0, white [no expression] to 5, dark [high expression]) for eosinophil peroxidase (EPX), neutrophil elastase, mast cell tryptase, and IL-33 in epithelial cells, endothelial cells and vascular smooth muscle cells in COVID-19 pneumonia (*n* = 49 samples from 15 patients), non-COVID pneumonia (*n* = 15 samples from 10 patients) and non-pneumonia participants (*n* = 10 samples from 5 participants). Histology block (Histo #/block ID), sample identification (SID); lung tissue lobe (upper, U, lower, L; left lung, L; right lung, R). Not determined (ND).

Supplementary Table 10. RNA sequencing datasets from endothelial cells.

A – IL-33 stimulation

HUVEC_IL-33

B – IL -33 and tozorakimab

HUVEC_IL-33tozo

C – type 2 inflammation

Gene symbol	Gene name	logFC	adj p-value
IL4	Interleukin-4	2.5	0.0002
TSLP	thymic stromal lymphopoietin	0.2	0.9705
CCL26	Eotaxin-3	-0.8	0.5467
IL5	Interleukin-5	ND	-
IL13	Interleukin-13	ND	-
CCL11	Eotaxin-1	ND	-
CCL24	Eotaxin-2	ND	-

Differential gene expression changes (log fold change (FC); *p* value and adjusted *p* values) in HUVEC treated with 30 ng/mL IL-33^{red} *versus* vehicle (6 and 24 h) (A), and IL-33^{red} (30 ng/mL) and tozorakimab (10 μg/mL) *versus* IL-33^{red} and isotype antibody (6 and 24 h) (B). (C), Summary of gene expression of type II inflammatory markers (*IL4*, *TSLP*, *IL5*, *IL13*, *CCL11*, *CCL24*, *CCL26*) in HUVEC treated with 30 ng/mL IL-33^{red} *versus* vehicle (log FC and fold change). Not detected (ND).

Supplementary Table 11. Endothelial IL-33^{red} signatures associate with infectious disease and lung injury signatures.

Disease Signature	Connectivity normScore	adj-p-value	Reference	Source
Acute Lung Injury	-3.48	9.64E-26	GSE1871	Mouse Lung Tissue
silicosis	-3.39	6.64E-23	GSE29110	Rat lung
Acute Lung Injury	-3.03	5.07E-18	GSE1871	Mouse Lung Tissue
pulmonary tuberculosis	-2.59	5.84E-06	GSE48027	Mouse Lung
severe combined immunodeficiency	-2.40	4.23E-07	GSE3414	Mouse Lung Tissue
pulmonary fibrosis	-2.07	1.51E-05	GSE2640	Mouse Tissue
pulmonary tuberculosis	-1.98	5.27E-04	GSE48027	Mouse Lung
pulmonary fibrosis	-1.81	5.02E-04	GSE2640	Mouse Lung Tissue
swine influenza	-1.71	1.86E-03	GSE48466	Human primary lung bronchial epithelial cells
nonspecific interstitial pneumonia	-1.24	1.76E-01	GSE5774	Human Lung Tissue
nonspecific interstitial pneumonia	-1.10	2.78E-01	GSE5774	Human Lung Tissue
Ventilator-associated lung injury	-1.03	2.08E-01	GSE2411	Mouse Lung Tissue
Pseudomonas Infection	-0.87	3.88E-01	GSE923	Human Lung Tissue
pulmonary tuberculosis	-0.81	3.42E-01	GSE48027	Mouse Lung
Pseudomonas Infection	-0.45	3.88E-01	GSE923	Human Lung Tissue
Ventilator-associated lung injury	-0.26	3.96E-01	GSE2411	Mouse Lung Tissue
Lung Injury	-0.02	5.45E-01	GSE1541	Human Lung Tissue

Summary of associations of HUVEC gene signatures with infectious disease and lung injury signatures. Adjusted p values (adj p value < 0.05) are shown above the bold line); Gene set enrichment (GSE) data sources.

Supplementary Table 12. List of ISARIC4C investigators

iSARIC-4c_list.xlsx
